# Hepatic gluconeogenesis and PDK3 upregulation drive cancer cachexia in flies and mice

**DOI:** 10.1038/s42255-025-01265-2

**Published:** 2025-04-16

**Authors:** Ying Liu, Ezequiel Dantas, Miriam Ferrer, Ting Miao, Mujeeb Qadiri, Yifang Liu, Aram Comjean, Emma E. Davidson, Tiffany Perrier, Tanvir Ahmed, Yanhui Hu, Marcus D. Goncalves, Tobias Janowitz, Norbert Perrimon

**Affiliations:** 1 Department of Genetics, Blavatnik Institute, Harvard Medical School, Boston, MA USA; 2 Department of Medicine, New York University Grossman School of Medicine, New York, NY USA; 3 Cold Spring Harbor Laboratory, Cold Spring Harbor, New York, NY USA; 4 Department of Medicine, Weill Cornell Medicine, New York, NY USA; 5 Northwell Health Cancer Institute, Northwell Health, New Hyde Park, New York, NY USA; 6 Howard Hughes Medical Institute, Boston, MA USA; 7 Present Address: Ohio State University College of Medicine, Columbus, OH USA

**Keywords:** Drosophila, Cancer, Metabolism, Cell signalling

## Abstract

Cachexia, a severe wasting syndrome characterized by tumour-induced metabolic dysregulation, is a leading cause of death in people with cancer, yet its underlying mechanisms remain poorly understood. Here we show that a longitudinal full-body single-nuclei-resolution transcriptome analysis in a *Drosophila* model of cancer cachexia captures interorgan dysregulations. Our study reveals that the tumour-secreted interleukin-like cytokine Upd3 induces fat-body expression of *Pepck1* and *Pdk*, key regulators of gluconeogenesis, disrupting glucose metabolism and contributing to cachexia. Similarly, in mouse cancer cachexia models, we observe IL-6–JAK–STAT-signalling-mediated induction of *Pck1* and *Pdk3* expression in the liver. Increased expression of these genes in fly, mouse, and human correlates with poor prognosis, and hepatic expression of *Pdk3* emerges as a previously unknown mechanism contributing to metabolic dysfunction in cancer cachexia. This study highlights the conserved nature of tumour-induced metabolic disruptions and identifies potential therapeutic targets to mitigate cachexia in people with cancer.

## Abstract (web summary)

In *Drosophila* and mouse models of cancer cachexia, Liu et al. show that tumour-derived cytokines induce hepatic gluconeogenesis, which in turn leads to dysregulation of metabolism and drives cancer cachexia.

## Main

More than 40% of people with cancer have cachexia, a life-threatening tumour-driven condition whose symptoms include massive weight loss, general inflammation, weakness, and fatigue^1^. A prominent driving force of these cachectic symptoms is the metabolic dysregulation stimulated by tumours, such as the systemic reprogramming of glucose metabolism^2^. In fact, glucose intolerance, characterized by the body’s reduced ability to effectively use glucose, is the earliest metabolic abnormality observed in people with cancer, and has been previously associated with insulin resistance^3–5^. Because insulin signalling is required for retaining glucose intake and glycolysis in peripheral tissues, people with cancer who have reduced insulin sensitivity could have a declined rate of glucose degradation, leading to glucose intolerance^6^.

Increased hepatic glucose production (gluconeogenesis) can also result in glucose intolerance^7^. Although the association between increased hepatic gluconeogenesis and cancer cachexia has been recognized for decades^8,9^, the underlying mechanism of pathogenesis is less understood. Gluconeogenesis requires a number of enzymes, such as phosphoenolpyruvate carboxykinase (PEPCK), pyruvate carboxylase, fructose 1,6-bisphosphatase (FBP), and glucose 6-phosphatase (G6P), to synthesize glucose from oxaloacetate through a series of reactions^10^. Among these enzymes, PEPCK is rate-limiting, catalysing the initial step of gluconeogenesis in which oxaloacetate is converted into phosphoenolpyruvate for glucose production^11^. Notably, tumour-bearing mice and rats with cachexia display increased hepatic expression of PEPCK^12,13^, suggesting its potential role in cachexia. An important physiological characteristic of gluconeogenesis is that it consumes energy, rather than producing it^14^. Thus, increased levels of hepatic gluconeogenesis in people with cancer might contribute to their loss of energy and weight^15,16^. Despite these observations, how hepatic gluconeogenesis activation in people with cancer is stimulated remains poorly understood. One model suggests that gluconeogenesis is activated through the Cori cycle by lactate derived from tumours^17^. In this model, tumours consume glucose and produce lactate through glycolysis, which is then released into the bloodstream. The liver takes up the lactate and converts it back into glucose through gluconeogenesis. This newly synthesized glucose is subsequently transported back to the tumours, where it fuels further lactate production, thus perpetuating the cycle. It has also been suggested that reduced insulin signalling activity might promote gluconeogenesis^18,19^. Further, inflammatory factors have also been proposed to play a role in the progression of insulin resistance^20^. For example, administration of the anti-inflammatory thiol compound pyrrolidine dithiocarbamate (PDTC) can inhibit aberrant hepatic PEPCK induction in a mouse model of intestinal cancer (*Apc*^*Min*/+^)^12^, indicating a role for inflammation in tumour-induced gluconeogenesis. A particularly relevant inflammatory cytokine is interleukin-6 (IL-6), which activates the JAK–STAT signalling pathway and has been shown to positively correlate with weight loss and mortality in people with cancer^21,22^. However, the effect of IL-6 on gluconeogenesis is controversial. For instance, IL-6 can stimulate gluconeogenesis in primary rat hepatocyte culture^23^, and is required to increase hepatic gluconeogenesis during acute stress^24^. Further, injection of IL-6, as well as prolonged exercise, which induces IL-6 expression, can upregulate hepatic PEPCK expression^25^. By contrast, liver-specific STAT3 deficiency in mice increases gluconeogenic gene expression, and IL-6–STAT3 signalling attenuates the induction of PEPCK and G6P by dexamethasone and cAMP in primary cultured mouse hepatocytes^26^. In addition, in a mouse model of obesity, amlexanox treatment induces IL-6 expression, which represses the expression of gluconeogenic genes^27,28^. Notably, in some cases, the effect of IL-6 on gluconeogenesis was not observed^29,30^. These discrepancies could be attributed to the dynamic and multifaceted regulation of PEPCK under varying conditions, potentially involving both antagonistic and synergistic interactions of different regulators. Altogether, these findings suggest that the regulation of gluconeogenesis by IL-6–JAK–STAT signalling is highly context-dependent and warrants further investigation.

In addition to enzymes directly involved in gluconeogenesis, pyruvate dehydrogenase kinase (PDK) might have a crucial role in promoting gluconeogenesis^31,32^. When gluconeogenesis is activated, PDK inhibits the conversion of pyruvate to acetyl-CoA by inactivating the pyruvate dehydrogenase complex (PDC), thereby redirecting pyruvate to pyruvate carboxylase for oxaloacetate production and gluconeogenesis^10,32^. Humans and rodents have four PDKs; in rodents, these are encoded by *Pdk1*–*Pdk4*, with different expression patterns across tissues^33,34^. In the rat liver, *Pdk2* is abundant, and *Pdk4* is expressed at a much lower level^34^. In mice, *Pdk1* and *Pdk2* are both highly expressed in the liver^35^. *Pdk2* and *Pdk4* show increased expression in the liver in response to starvation or diabetes^36–38^. Notably, *Pdk3* expression was nearly undetectable in the liver in humans and rodents, whether under normal, starvation, or diabetic conditions^34,35,39^, indicating a distinct regulatory mechanism and physiological role of *Pdk3*. In support of this notion, PDK3 displays many unusual biochemical characteristics: (1) among the recombinant PDK isoenzymes, PDK3 exhibits the highest catalytic activity, 25-fold higher than the activity of PDK2 (ref. ^34^); (2) activation of PDK3 does not depend on the levels of NADH and acetyl-CoA, which are required for activation of other PDKs^34^; and (3) among the four PDKs, PDK3 is least sensitive to the inhibition of pyruvate, and 40-fold less sensitive than PDK2 for this feedback inhibition^34,40^. Despite observations of these notable biochemical characteristics and a potentially substantial contribution to hepatic gluconeogenesis, how PDK3 is regulated in the liver remains unknown.

In recent years, several models of organ wasting and cachexia have emerged in *Drosophila*^41,42^. In particular, expression of an activated form of the *Yorkie* oncogene (*Yki*, also known as *Yap*) in adult intestinal stem cells (ISCs) (*esg>yki*^act^) generates tumours associated with cachectic properties^43^. These tumours secrete at least four factors: ecdysone-inducible gene L2 (ImpL2), PDGF- and VEGF-related factor 1 (Pvf1), ion transport peptide (ITP), and unpaired 3 (Upd3)^43–46^. ImpL2 antagonizes insulin signalling, leading to reduced anabolism in peripheral tissues^43,45^. Pvf1, a cytokine reminiscent of PDGF and VEGF, activates ERK signalling in peripheral tissues, such as muscle and the fat body, a crucial fly tissue whose metabolic functions are analogous to those of the human liver. This triggers catabolism while stimulating renal JNK signalling, leading to kidney dysfunction^44,47^. Similarly, the isoform F of ITP (ITP_F_), a fly antidiuretic hormone, impairs renal function, which causes accumulation of body fluid^46^. Finally, Upd3, the fly orthologue of IL-6, induces *ImpL2* expression in peripheral tissues, impairing insulin signalling and contributing to body wasting^45^. Given that cancer cachexia is a multi-organ syndrome, we performed single-nuclei RNA sequencing (snRNA-seq) of the full body of flies with *yki*^act^ gut tumours. Our transcriptomic analysis, combined with metabolic profiling, revealed that tumour-secreted Upd3 regulates JAK–STAT activity and promotes *Pepck1* and *Pdk* expression in the adipose tissue, the fly fat body. This parallels the activation of hepatic gluconeogenesis, given that the fat body performs many liver-like functions in flies. Importantly, using both mouse models of cancer cachexia and data from healthy individuals and people with cancer, we identified a conserved pathogenetic role for IL-6-induced hepatic expression of *Pck1* and *Pdk3*. Altogether, our findings could pave the way to a potential therapeutic strategy of targeting hepatic gluconeogenesis and PDK3 in IL-6-related cancer cachexia.

## Results

### Body-wide gene expression and cell proportion of Yki flies

**Fig. 1:**
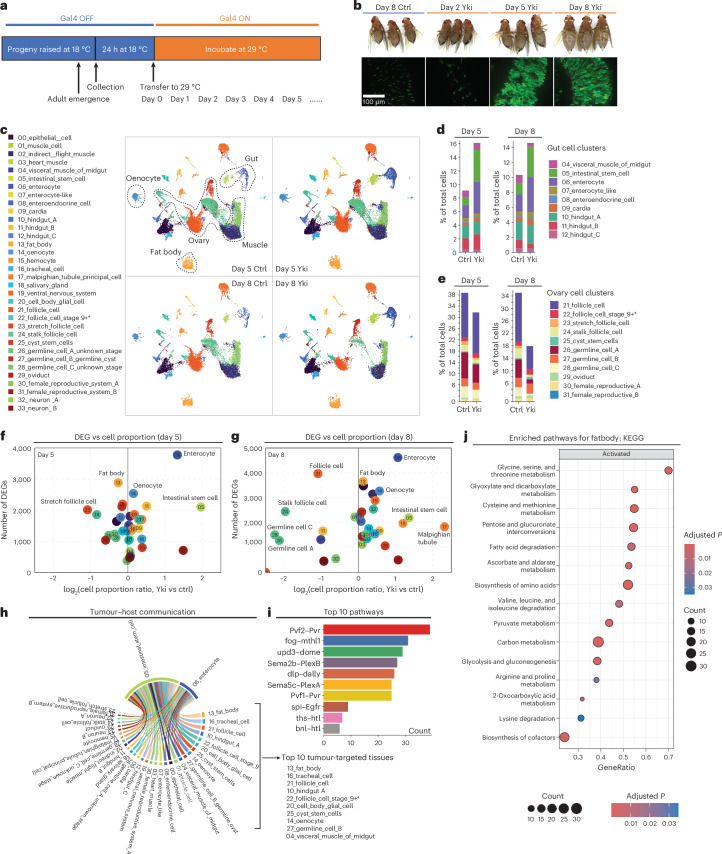
Full-body single-nucleus transcriptome survey of Yki flies. **a**, The experimental design for gut tumour induction in flies. **b**, Representative images of gut tumours and phenotypes of Yki flies at days 2, 5, and 8, and control (Ctrl) flies at day 8. The experiment was repeated three times independently, producing similar results. **c**, A UMAP visualization of cell clusters of control and Yki flies at days 5 and 8, revealed by snRNA-seq. **d**,**e**, Bar plot showing the change in total cell percentage of gut cells (**d**) and ovary cells (**e**) between control and Yki flies at days 5 and 8; cell proportions were normalized to each condition. **f**,**g**, A scatter plot showing the change in cell proportions (Yki versus Ctrl) at day 5 (**f**) and day 8 (**g**) on the *x* axis, and the number of DEGs, which were defined as having *P* < 0.05 and absolute log_2_-transformed fold change > 0.38 on the *y* axis, calculated by the Wilcoxon rank-sum test. **h**, FlyPhone analysis was performed to calculate the interaction scores between tumour cells (05_intestinal_stem_cell and 06_enterocyte) and peripheral tissues of various ligand–receptor pairs involved in cell–cell communication, persisting at both days 5 and 8. **i**, The top 10 pathways between tumour and host tissues identified by FlyPhone. **j**, GSEA was performed on fat body cells (cluster 13_fat_body), and the top 15 enriched pathways are shown. GeneRatio represents the number of input genes mapped to a given pathway divided by the total number of input genes. Statistical significance was assessed by multiple comparisons using the Benjamini–Hochberg method. KEGG, Kyoto Encyclopedia of Genes and Genomes. See also Extended Data Fig. 1.

**Extended Data Fig. 1:**
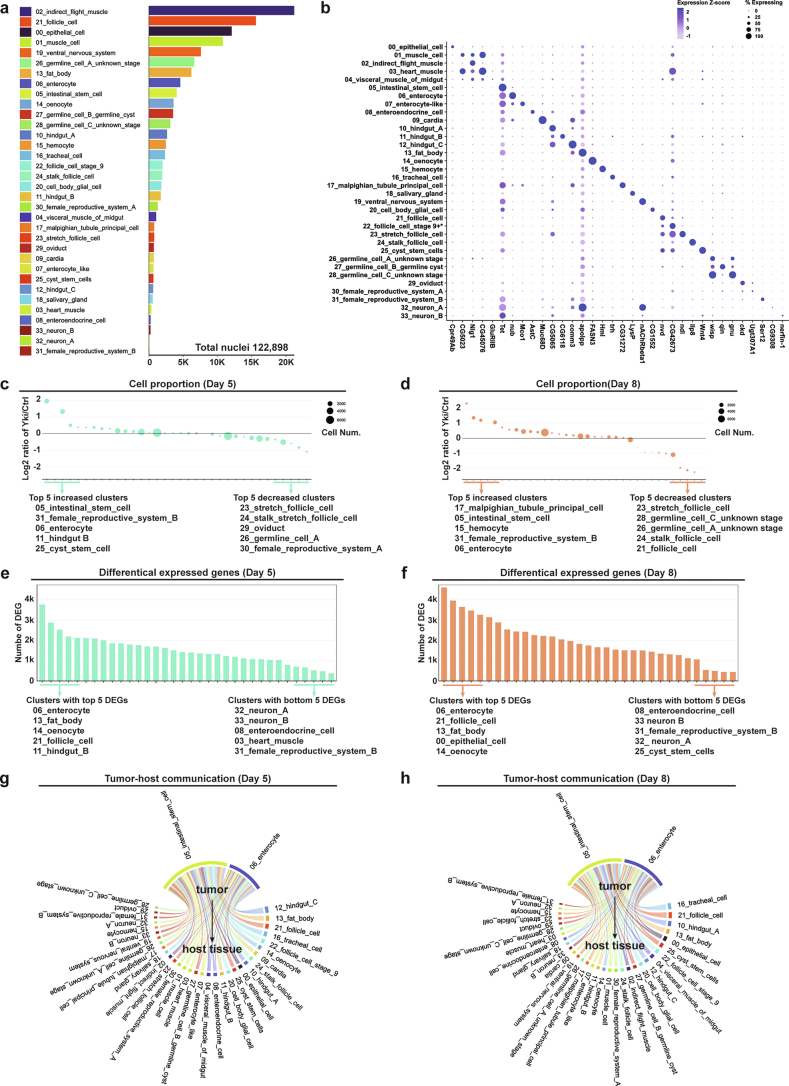
Related to Figure 1. . **(a)** Total number of nuclei detected in each cell cluster across all samples retrieved from snRNA-seq. **(b)** Dot plot showing marker genes of each cell cluster. Color scale indicates Z-score, and size indicates expression percentage. Dot plot showing the change in cell proportion of each cell cluster between Yki and control at Day 5 **(c)** and Day 8 **(d)**. Cell proportions were normalized to each condition and the Log2 ratio Yki vs control cell counts were calculated. The size of each dot represents the number of cells in Yki flies, and the plot is ordered by descending Log2 ratio. Bar plots showing the number of DEGs in each cluster (using a Wilcoxon Rank Sum test with p-value < 0.05 and abs (Log2FC) > 0.38) at Day 5 **(e)** and Day 8 **(f)**. The top and bottom five clusters are labelled. Flyphone prediction of cell-to-cell communication at Day 5 **(g)** and Day 8 **(h)**. The interaction scores of tumor cells (05_intestinal_stem_cell and 06_enterocyte) and peripheral tissues was calculated. The difference in interaction score between Yki and control was taken and ligand-receptor pairs which did not express a DEG in either the tumor or host tissue between Yki and control were filtered out. The width of each connection reflects the number of ligand-receptor pairs between tumor and host.

To achieve a comprehensive understanding of tumour-induced wasting in host organs, we examined the full body transcriptome of flies with *yki*^act^ gut tumours (*esg>yki*^act^; referred to as Yki flies) at single-nuclei resolution. Yki flies develop tumours at day 2 after induction of *yki*^act^ expression, and these tumours encompass most of the gut at day 5 (Fig. 1a,b). Wasting of peripheral organs and bloating (accumulation of body fluid) in these animals starts at day 5 and become severe at day 8 (ref. ^44^) (Fig. 1a,b). To characterize the transcriptional changes occurring in peripheral tissues, we isolated nuclei from Yki flies at days 5 and 8, along with the appropriate controls, and performed snRNA-seq. Heads were excluded from these samples because our study focused on changes in the gut, muscle, fat body, adipose tissue, and oenocytes. In total, 122,898 nuclei were profiled (25,146 from control and 42,375 from Yki flies at day 5; 19,050 from control and 36,327 from Yki flies at day 8), with a median of 559 genes and 990 unique molecular identifiers (UMIs) per nucleus across all conditions. We then generated a uniform manifold approximation and projection (UMAP) plot of all cells, identifying 34 cell clusters (Fig. 1c and Extended Data Fig. 1a,b). These clusters were annotated on the basis of the marker genes of *Drosophila* cell types reported by the Fly Cell Atlas^48^ (Extended Data Fig. 1a,b), and they represent cells from all the major body organs (Fig. 1c).

We assessed alterations in host organs using two measures: the number of differentially expressed genes (DEGs) and changes in cell numbers. The expression of *yki*^act^ in ISCs increases cell proliferation. In line with this, a higher proportion of ISC nuclei were recovered in Yki flies than in controls (Fig. 1d and Extended Data Fig. 1c,d). In addition, the proportion of enterocyte nuclei was also increased, suggesting that *yki*^act^ ISCs are still capable of differentiation (Fig. 1d and Extended Data Fig. 1c,d). ISCs and enterocytes are among the top five increased cell clusters at both days 5 and 8 (Fig. 1d and Extended Data Fig. 1c,d). Conversely, there was a notable decrease in many ovarian cell clusters, particularly at day 8, including several germline and follicle cell clusters (Fig. 1e and Extended Data Fig. 1c,d), which is consistent with the previous observation that *yki*^act^ gut tumours lead to ovarian atrophy^43^. Disturbed homeostasis in host tissues, such as hyperproliferation and degeneration, can lead to substantial changes in gene expression. For instance, the enterocyte cluster, which exhibits one of the largest increases in cell proportion, showed the highest number of DEGs (Fig. 1f,g, and Extended Data Fig. 1e,f). Notably, we observed that fat-body cells displayed a large number of DEGs at both days 5 and 8, despite only minor changes in their proportion (Fig. 1f,g and Extended Data Fig. 1e,f), suggesting a transcriptomic shift that is independent of cell proliferation or tissue atrophy, pointing to disruptions of its physiological function.

To decipher how tumours induce organ wasting, we analysed tumour–host organ communication in Yki flies using FlyPhone, an integrated web-based resource for predicting cell–cell communication in *Drosophila*^49^. On the basis of the expression levels of ligand–receptor pairs, we generated a list of pathways potentially mis-regulated between Yki tumours (ISCs and enterocytes) and peripheral organs and tissues on days 5 and 8 (Extended Data Fig. 1g,h). Given that the wasting phenotypes of Yki flies continue to develop after day 5, perturbed communications that are not consistent from day 5 to day 8 are unlikely to contribute to these phenotypes. Therefore, we focused on the interactions that persist at both days 5 and 8 (Fig. 1h). The top tumour-to-host organ communications include PDGF–VEGF signalling and Upd3–JAK–STAT signalling (Fig. 1i). Notably, the fat body emerged as the primary target tissue of gut tumours, as indicated by the number of ligand–receptor interactions (Fig. 1h). Gene set enrichment analysis revealed enriched activity in glycolysis, gluconeogenesis, fatty acid metabolism, and amino acid metabolism in the fat body in general (Fig. 1j). Considering that cancer cachexia is driven by metabolic disorders^50^ and that no significant changes were observed in the proportion of fat-body cells, our data—particularly the large number of DEGs and disrupted communication signalling in the fat body—suggest that its physiological aberrations, which are likely to be related to metabolism, play a key role in the cachectic phenotypes observed in Yki flies.

### Reprogramming of fat-body metabolism by the tumourous gut

**Fig. 2:**
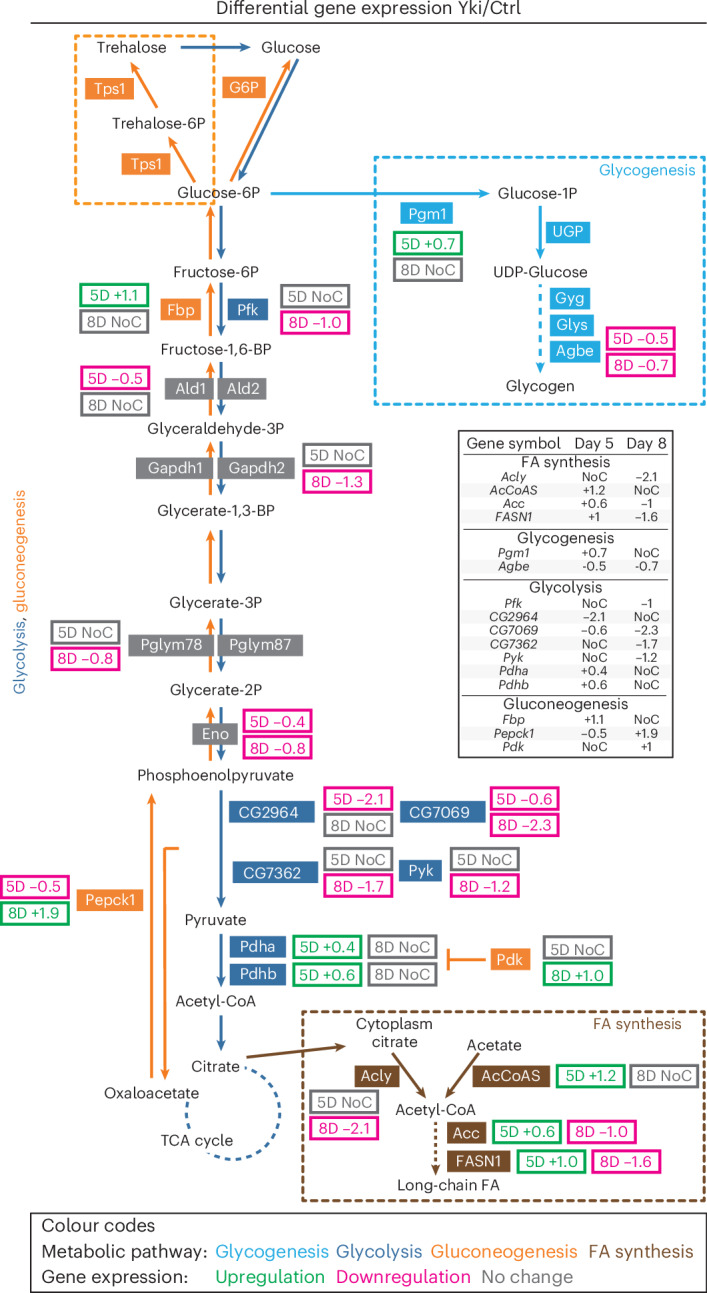
Fat-body metabolic pathway profiling. The relative expression (Yki versus Ctrl, average log_2_-transformed fold change) at days 5 (5D) and 8 (8D) of genes involved in glycolysis, gluconeogenesis, glycogenesis, and fatty acid (FA) synthesis. Shared enzymes of glycolysis and gluconeogenesis are shown in grey; upregulation is indicated by green, downregulation is indicated by magenta, and no significant change is indicated in grey (NoC). See also Extended Data Fig. 2.

**Extended Data Fig. 2:**
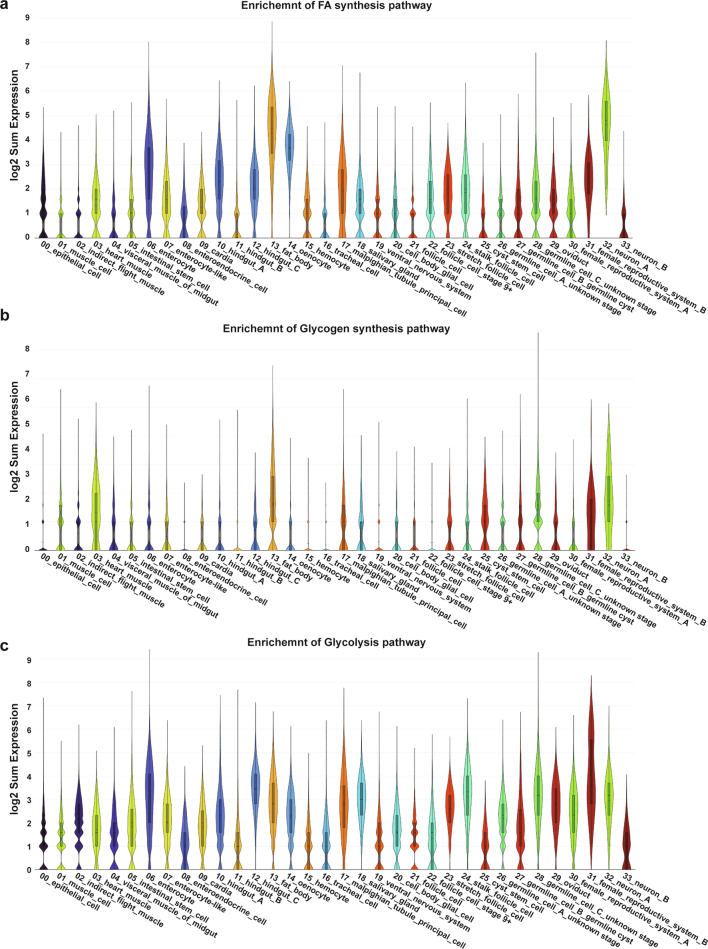
Related to Figure 2. . Total expression of fatty acids synthesis pathway genes **(a)**, glycogen synthesis pathway genes **(b)**, and glycolysis pathway genes **(c)** in each cell cluster. Metabolic pathway genes are retrieved from Kyoto Encyclopedia of Genes and Genomes (KEGG). Fatty acids synthesis pathway genes include Acsl, beg, bgm, CG12170, CG16935, CG3603, CG3961, FASN1, hll, ATPCL, AcCoAS, ACC. Glycogen synthesis pathway genes include AGBE, GlyS, Gyg, Pgm1, Pgm2a, Pgm2b, UGP. Glycolysis pathway genes include Ald1, Ald2, CG12229, CG2964, CG6650, CG7059, CG7069, CG7362, CG9010, CG9961, Eno, Gapdh1, Gapdh2, Hex-A, Hex-C, Hex-t1, Hex-t2, Pfk, Pgi, Pgk, Pglym78, Pglym87, PyK, Tpi. (00 _epithelial_cell n=12066, 01_muscle_cell n=10791, 02_indirect_flight_muscle n=21190, 03_heart_muscle n=381, 04_visceral_muscle_of_midgut n=1064, 05 _intestinal_stem_cell n=4066, 06_enterocyte n=4583, 07 _enterocyte-like n=674, 08_enteroendocrine_cell n=264, 09_cardia n=715, 10_hindgut_A n=2650, 11_hindgut_B n=1732, 12_hindgut_C n=621, 13_fat_body n=6200, 14_oenocyte n=3597, 15_hemocyte n=2468, 16 _tracheal _cell n=2344, 17_malpighian_tubule_principal_cell n=780, 18_salivary_gland n=562, 19_ventral_nervous_system n=7585, 20_cell_body _glial_cell n=1827, 21_follicle_cell n=15600, 22_follicle_cell _stage 9+* n=1994, 23 _stretch_follicle_cell n=779, 24 _stalk_follicle_cell n=1954, 25_cyst_stem_cells n=662, 26_germline_cell_A_unknown stage n=6615, 27_germline_cell_B_germline cyst n=3518, 28 _germline_cell_C_unknown stage n=3130, 29_oviduct n=723, 30_female_reproductive_system_A n=1294, 31_female_reproductive_system_B n=48, 32_neuron_A n=77, 33_neuron_B n=236). Box plots denote the medians, means, and the interquartile range. The whiskers of each box plot are the lowest and highest expression levels.

Our previous study revealed reduced levels of energy storage (lipids and glycogen) and increased glucose in Yki flies^43^, prompting us to examine relevant metabolic pathways in the fat body. Indeed, we observed enrichments of genes involved in fatty acid synthesis and glycogen synthesis in the fat-body cell cluster (Extended Data Fig. 2a,b). Glycolysis genes, being fundamental to energy generation across different cell types, were more evenly expressed across all clusters (Extended Data Fig. 2c). Next, we profiled these metabolic pathways at both time points (Fig. 2). Fatty acid synthesis in *Drosophila* requires several key enzymes, including ATP citrate lyase (Acly), acetyl coenzyme A synthase (AcCoAS), acetyl-CoA carboxylase (ACC), and fatty acid synthase 1 (FASN1)^51,52^. Notably, the expression of these genes indicated distinct changes in fatty acid synthesis between day 5 and day 8. At day 5, fatty acid synthesis was upregulated, as indicated by the increased expression of *AcCoAS*, *ACC*, and *FASN1* (Fig. 2). However, at day 8, there was a marked decrease in fatty acid synthesis, reflected by the downregulation of *Acly*, *ACC*, and *FASN1* (Fig. 2). This observation is consistent with the more-pronounced wasting phenotypes seen at day 8. Additionally, glycogen synthesis levels were reduced, particularly at day 8, as evidenced by the downregulation of 1,4-alpha-glucan branching enzyme (encoded by *Agbe*) (Fig. 2), which is required for glycogen synthesis^53^. Glycolysis involves enzymes encoded by *Pfk*, *Gapdh2*, and *Eno*, and a number of pyruvate kinases (encoded by *CG7069*, *CG7362* and *Pyk*)^54–57^. At both time points, glycolysis was reduced, with a more severe downregulation of these enzymes at day 8 (Fig. 2). This could be attributable to the known systemic inhibition of insulin signalling in Yki flies^43^. In addition, we observed notable upregulation of *Pepck1* and *Pdk* at day 8 (Fig. 2), both of which are involved in gluconeogenesis^11,31,58^.

### Fat-body gluconeogenesis increases trehalose levels in Yki flies

**Fig. 3:**
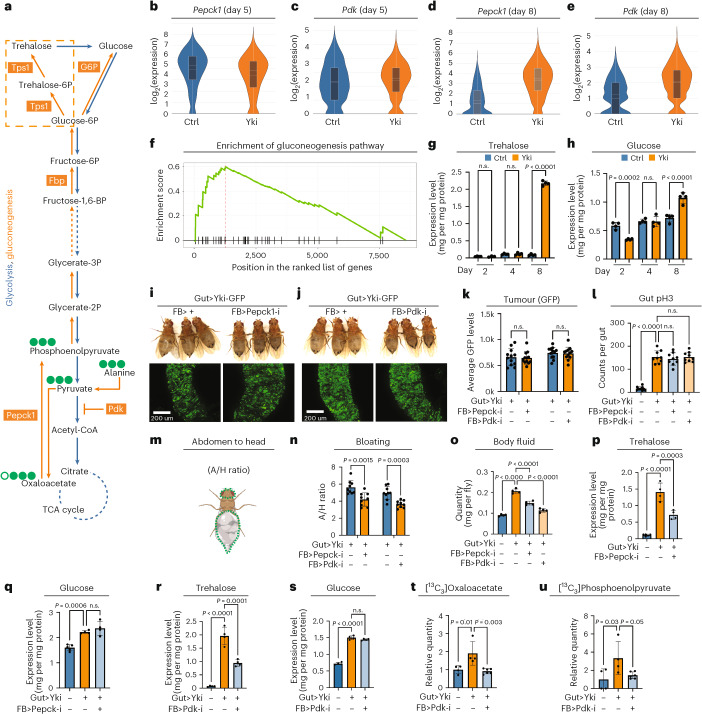
Upregulation of *Pepck1* and *Pdk* in the fat body of Yki flies stimulates gluconeogenesis. **a**, A schematic of the gluconeogenesis pathway in *Drosophila*. **b**,**c**, Violin plots of expression levels of *Pepck1* (**b**) and *Pdk* (**c**) at day 5 in the fat-body cell cluster; data were retrieved from snRNA-seq (Ctrl *n* = 1,263, Yki *n* = 1,788). Box plots show the medians (top centre line), means (bottom centre line), and the interquartile ranges. The whiskers of each box plot extend to the lowest and highest expression levels. **d**,**e**, Violin plots of expression levels of *Pepck1* (**d**) and *Pdk* (**e**) at day 8 in the fat-body cell cluster retrieved from snRNA-seq (Ctrl *n* = 1,022, Yki *n* = 2,127). In the Ctrl group, box plots show the mean (top centre line) and median (bottom centre line); in the Yki group, the medians are represented by the top centre line and the means by the bottom centre line. Box plots also display interquartile ranges. The whiskers of each box plot extend to the lowest and highest expression levels. **f**, GSEA of the gluconeogenesis pathway, performed on fat-body genes retrieved from snRNA-seq. **g**,**h**, Whole-body trehalose (**g**) and glucose (**h**) levels at various tumour-induction time points (2, 4, and 8 days) (*n* = 4). **i**,**j**, Representative images of gut tumours and the phenotype of Yki flies with or without fat-body *Pepck1* (**i**) and *Pdk* (**j**) depletion at day 6. **k**, Quantification of gut tumours (GFP signal) of Yki flies with or without fat-body *Pepck1* and *Pdk* depletion at day 6 (*n* = 12). **l**, Total pH3 counts per gut of Yki flies with and without fat-body *Pepck1* and *Pdk* depletion at day 6 (*n* = 9). **m**,**n**, The bloating rate quantification method (**m**) and results (**n**), represented by the abdomen to head size (A/H) ratio of Yki flies with or without fat-body *Pepck1* and *Pdk* depletion at day 6 (*n* = 9). **o**, Body fluid levels of Yki flies with or without fat body *Pepck1* and *Pdk* depletion at day 6 (*n* = 4). **p**,**q**, Whole-body trehalose (**p**) and glucose (**q**) levels of control flies, Yki flies, and Yki flies with fat-body *Pepck1* depletion at day 6 (Ctrl *n* = 5, Yki *n* = 4, Yki with fat-body *Pepck1* depletion *n* = 4). **r**,**s**, Whole-body trehalose (**r**) and glucose (**s**) levels of control flies, Yki flies, and Yki flies with fat-body *Pdk* depletion at day 6 (*n* = 4). **t**,**u**, Relative quantities of [^13^C_3_]oxaloacetate (**t**) and [^13^C_3_]phosphoenolpyruvate (**u**) in control flies, Yki flies, and Yki flies with fat-body *Pdk* depletion at day 6 (Ctrl *n* = 4, Yki *n* = 4, Yki with fat-body *Pdk* depletion *n* = 6). Statistical significance was assessed using unpaired two-sided Student’s *t*-test (**k**,**n**) and ordinary one-way ANOVA (**g**,**h**,**l**,**o**–**u**). The error bars indicate the s.d., with the mean at the centre. **m** was created in BioRender (https://BioRender.com/w65y109). See also Extended Data Fig. 3. Source data

**Extended Data Fig. 3:**
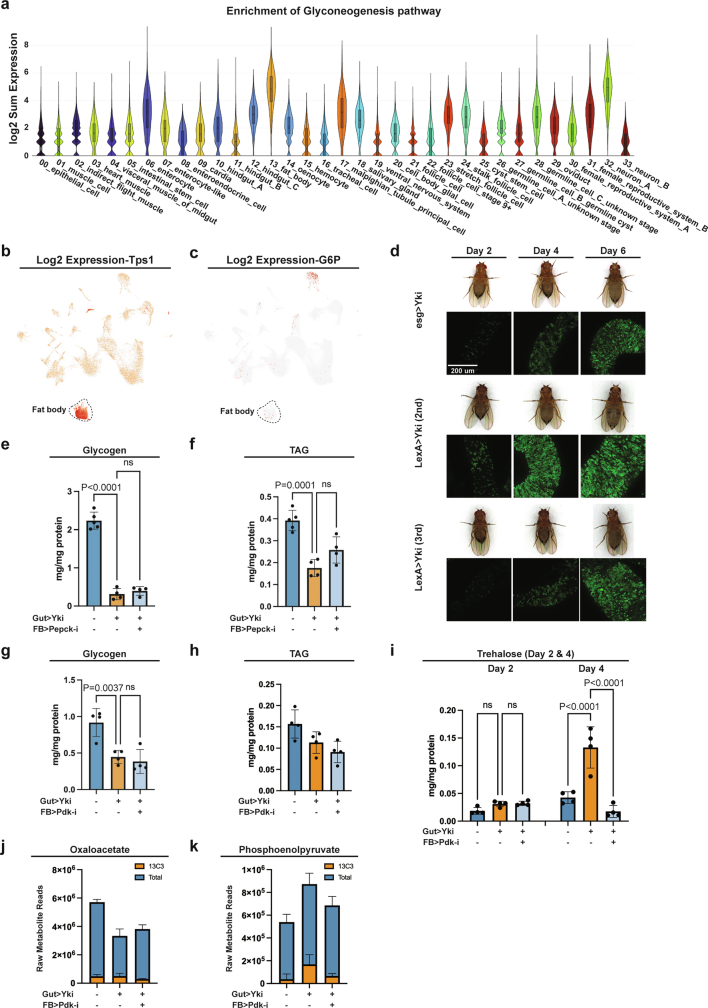
Related to Figure 3. . **(a)** Total expression of gluconeogenesis pathway genes in each cell cluster. Gluconeogenesis pathway genes are retrieved from Kyoto Encyclopedia of Genes and Genomes (KEGG), include Ald1, Ald2, CG7059, CG9010, CG9961, Eno, fbp, G6P, Gapdh1, Gapdh2, Pepck1, Pepck2, Pgk, Pglym78, Pglym87, Tpi, Tps1, Pdk (00 _epithelial_cell n=12066, 01_muscle_cell n=10791, 02_indirect_flight_muscle n=21190, 03_heart_muscle n=381, 04_visceral_muscle_of_midgut n=1064, 05 _intestinal_stem_cell n=4066, 06_enterocyte n=4583, 07 _enterocyte-like n=674, 08_enteroendocrine_cell n=264, 09_cardia n=715, 10_hindgut_A n=2650, 11_hindgut_B n=1732, 12_hindgut_C n=621, 13_fat_body n=6200, 14_oenocyte n=3597, 15_hemocyte n=2468, 16 _tracheal _cell n=2344, 17_malpighian_tubule_principal_cell n=780, 18_salivary_gland n=562, 19_ventral_nervous_system n=7585, 20_cell_body _glial_cell n=1827, 21_follicle_cell n=15600, 22_follicle_cell _stage 9+* n=1994, 23 _stretch_follicle_cell n=779, 24 _stalk_follicle_cell n=1954, 25_cyst_stem_cells n=662, 26_germline_cell_A_unknown stage n=6615, 27_germline_cell_B_germline cyst n=3518, 28 _germline_cell_C_unknown stage n=3130, 29_oviduct n=723, 30_female_reproductive_system_A n=1294, 31_female_reproductive_system_B n=48, 32_neuron_A n=77, 33_neuron_B n=236). Box plots denote the medians, means, and the interquartile range. The whiskers of each box plot are the lowest and highest expression levels. UMAP visualization of expression levels of *Tps1* **(b)** and *G6p* **(c)** across all cell clusters retrieved from snRNA-seq. **(d)** Representative gut tumor and phenotypes of Yki flies induced by indicated methods (*esg-Gal4>UAS-Yki, esg-LexA>LexAop-Yki 2*^*nd*^, and *esg-LexA>LexAop-Yki 3*^*rd*^). The experiment was repeated two times independently, producing similar results. Whole-body glycogen **(e)**, TAG **(f)**, levels of control flies, Yki flies, and Yki flies with fat body *Pepck1* depletion at Day 6 (n=4). Whole-body glycogen **(g)**, TAG **(h)**, levels of control flies, Yki flies, and Yki flies with fat body *Pdk* depletion at Day 6 (n=4). **(i)** Whole-body trehalose levels of control flies, Yki flies, and Yki flies with fat body *Pdk* depletion at Day 2 and Day 4 (n=4). Raw metabolite reads and normalized proportions of ^13^C3 labeled oxaloacetate **(j)** and phosphoenolpyruvate **(k)** (Ctrl n=4, Yki n=4, Yki with fat body *Pdk* depletion n=6). Statistical significance was assessed by ordinary one-way ANOVA (**e**-**g**, **i**). ns indicates not significant. Error bars indicate SDs with the mean as the center. Source data

*Pepck1*, which encodes the rate-limiting enzyme of gluconeogenesis, is specifically upregulated on day 8, indicating a possible increase in gluconeogenesis at this later stage (Fig. 3a,b,d). Additionally, the upregulation of *Pdk* at day 8 might synergize with *Pepck1* to further enhance gluconeogenesis (Fig. 3a,c,e). In a previous study, we reported increased levels of trehalose and glucose in Yki flies, which we initially attributed to reduced glycolysis caused by inhibition of insulin signalling^43^. However, our current findings suggest that increased gluconeogenesis in the fat body could also contribute to the abnormally high trehalose and glucose levels observed in Yki flies. Supporting this hypothesis, we detected an enrichment of gluconeogenesis-related genes in the fat body across organs (Fig. 3f and Extended Data Fig. 3a). Moreover, significant increases in whole-body glucose and trehalose levels were detected in Yki flies only at day 8, coinciding with a marked increase in *Pepck1* and *Pdk* expression (Fig. 3g,h).

In mammals, G6P catalyses the final step of gluconeogenesis to produce glucose. In flies, however, its role is fulfilled by trehalose-6-phosphate synthase 1 (Tps1), which generates trehalose instead of glucose^59,60^ (Fig. 3a). Consistent with this, we observed specific expression of *Tps1*, but not *G6P*, in the fat-body cell cluster (Extended Data Fig. 3b,c), suggesting that trehalose is the end product of fat-body gluconeogenesis in flies. To further investigate the role of fat-body *Pepck1* and *Pdk* in regulating whole-body carbohydrate levels, we selectively decreased their expression in the fat body of Yki flies using the dual binary systems, GAL4–UAS and LexA–LexAop, to manipulate gene expression in the gut and fat body, respectively^61^. We generated two LexAop lines on the second and third chromosome, respectively, to express *yki*^act^. Notably, the second-chromosome LexAop-Yki line exhibited stronger tumour growth in the gut and severe bloating at day 6, earlier than the third-chromosome LexAop-Yki and UAS-Yki lines (Extended Data Fig. 3d). We timed our experiments on the basis of this observation. Using the LexAop-Yki line, we found that depletion of *Pepck1* and *Pdk* in the fat body of Yki flies did not result in changes in green fluorescent protein (GFP)-labelled gut tumour cells, but did suppress the bloating phenotype (Fig. 3i,j). Quantification of GFP signalling in gut tumours showed no differences between Yki flies and those with depletion of *Pepck1* or *Pdk* in the fat body (Fig. 3k). Additionally, immunostaining for phosphorylated histone 3 (pH3), a marker for mitotic cells, indicated no differences in the gut proliferation rates (Fig. 3l). Although no change was observed in gut tumours, the bloating phenotype and increased body fluid levels were significantly ameliorated in Yki flies with fat-body *Pepck1* or *Pdk* depletion (Fig. 3m–o), indicating that wasting was attenuated in these flies. Furthermore, their whole-body trehalose levels were significantly reduced (Fig. 3p,r), whereas levels of glucose and other analysed metabolites remained unaffected by *Pepck1* or *Pdk* depletion (Fig. 3q,s and Extended Data Fig. 3e–h). Notably, whole-body trehalose levels begin to increase at day 4 in Yki flies (second-chromosome line), coinciding with the formation of gut tumours, before bloating occurs (Extended Data Fig. 3d,i). This increase can be inhibited by depleting *Pdk* in the fat body, suggesting that the tumours induce fat-body gluconeogenesis before the onset of bloating, and that the accumulation of trehalose might contribute to the bloating phenotype in these flies (Extended Data Fig. 3i). To further confirm the role of gluconeogenesis in Yki flies, we traced ^13^C_3_-labelled alanine, the most gluconeogenic amino acid. As expected, we detected increased proportions of ^13^C_3_-labelled oxaloacetate and phosphoenolpyruvate in Yki flies, which were notably eliminated by fat body *Pdk* depletion (Fig. 3a,t–u and Extended Data Fig. 3j,k). Altogether, these findings suggest that fat-body gluconeogenesis, induced by the upregulation of *Pepck1* and *Pdk*, leads to increased trehalose levels in Yki flies.

### Increased gluconeogenesis is independent of insulin and glucagon

**Fig. 4:**
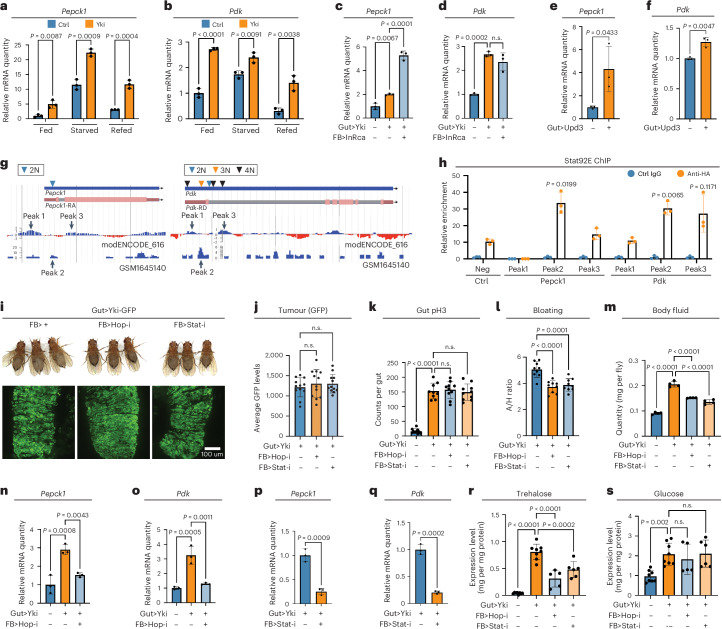
The JAK–STAT signalling pathway regulates *Pepck1* and *Pdk* expression in the fat body. **a**,**b**, Quantitative reverse transcription PCR (qRT–PCR) of *Pepck1* (**a**) and *Pdk* (**b**) mRNA in the fat body of fed, starved, and refed flies at day 6 (*n* = 3). **c**,**d**, *Pepck1* (**c**) and *Pdk* (**d**) mRNA in the fat body of control, Yki flies, and Yki flies with fat-body *InR-ca* (constitutive-activated form of InR) expression at day 8 (*n* = 3). **e**,**f**, *Pepck1* (**e**) and *Pdk* (**f**) mRNA in the fat body of flies with or without ISC *Upd3* expression at day 8 (*n* = 3). **g**, Data from the ChIP–seq database indicating enrichment of Stat92e binding at the *Pepck1* and *Pdk* gene regions. The transcript isoforms *Pepck1*-RA and *Pdk*-RD were used to indicate the gene regions. Inverted triangle, STAT-binding motif (2N, TTCNNGAA; 3N, TTCNNNGAA; 4N, TTCNNNNGAA). **h**, ChIP revealed the enrichment of haemagglutinin (HA)-tagged Stat92E binding at the *Pepck1* and *Pdk* gene regions, shown by fold changes relative to control IgG at day 8 (*n* = 3 biological replicates). Neg, negative control. **i**, Images of gut tumours and phenotypes of Yki flies with or without fat-body *hop* or *Stat92e* depletion at day 6. **j**, Quantification of gut tumours (GFP signal, arbitrary units) of Yki flies with or without fat-body *hop* or *Stat92e* depletion at day 6 (n = 12). **k**, Total pH3 counts per gut of control flies and Yki flies with or without fat-body *hop* or *Stat92e* depletion at day 6 (*n* = 10). **l**, Quantification of bloating phenotypes (A/H ratio) of Yki flies with or without fat-body *hop* or *Stat92e* depletion at day 6 (*n* = 9). **m**, Body-fluid levels of Yki flies with or without fat-body *hop* or *Stat92e* depletion at day 6 (*n* = 4). **n**,**o**, *Pepck1* (**n**) and *Pdk* (**o**) expression in the fat body of control, Yki flies, and Yki flies with fat-body *hop* depletion at day 6 (*n* = 3). **p**,**q**, *Pepck1* (**p**) and *Pdk* (**q**) expression in the fat body of Yki flies and Yki flies with fat-body *Stat92e* depletion at day 6 (*n* = 3). **r**,**s**, Whole-body trehalose (**r**) and glucose (**s**) levels of control (*n* = 8), Yki flies (*n* = 8), and Yki flies with fat-body *hop* (*n* = 5) or *Stat92e* (*n* = 6) depletion at day 6. Statistical significance was assessed using the unpaired two-sided Student’s *t*-test (**e**,**f**,**p**,**q**) and ordinary one-way ANOVA (**a**–**d**,**h**,**j**–**o**,**r**,**s**). Error bars indicate the s.d., with the mean as the centre. See also Extended Data Fig. 4. Source data

**Extended Data Fig. 4:**
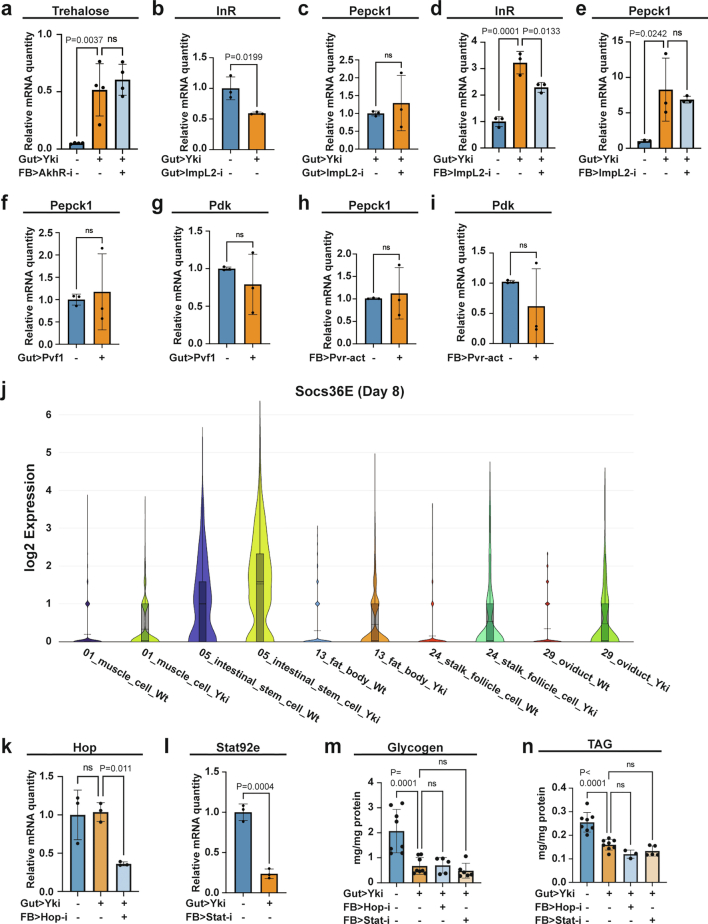
Related to Figure 4. . **(a)** Whole-body trehalose levels of control flies, Yki flies, and Yki flies with fat body *AkhR* depletion at Day 6 (n=4). qRT-PCR analysis of *InR* **(b)** and *Pepck1* **(c)** mRNA levels in the fat body of Yki flies without or with ISC *ImpL2* depletion at Day 8 (n=3). qRT-PCR analysis of *InR* **(d)** and *Pepck1* **(e)** mRNA levels in the fat body of Yki flies without or with fat body *ImpL2* depletion at Day 6 (n=3). qRT-PCR analysis of *Pepck1* **(f)** and *Pdk* **(g)** mRNA levels in fat body of flies with or without ISC *Pvf1* expression at Day 8 (n=3). qRT-PCR analysis of *Pepck1* **(h)** and *Pdk* **(i)** mRNA levels in fat body of flies without and with *Pvr-act* expression at Day 8 (n=3). **(j)** Socs36E expression in the muscle, ISC, fat body, stalk follicle cell, and oviduct cell clusters at Day 8 retrieved from snRNA-seq and visualized by violin plots. (01_muscle_cell_Wt n=1762, 01_muscle_cell_Yki n=3138, 05 _intestinal_stem_cell_Wt n=298, 05 _intestinal_stem_cell_Yki n=1459, 13_fat_body_Wt n=1022, 13_fat_body_Yki n=2127, 24 _stalk_follicle_cell_Wt n=567, 24 _stalk_follicle_cell_Yki n=276, 29_oviduct_Wt n=151, 29_oviduct_Yki n=169). Box plots denote the medians, means, and the interquartile range. The whiskers of each box plot are the lowest and highest expression levels. **(k)** qRT-PCR analysis of *hop* mRNA levels in fat body of control flies, Yki flies, and Yki flies with fat body *hop* depletion at Day 6 (n=3). **(l)** qRT-PCR analysis of *Stat92e* mRNA levels in fat body of Yki flies without or with fat body *Stat92e* depletion at Day 6 (n=3). Whole-body glycogen **(m)** and TAG **(n)** levels of control flies, Yki flies, Yki flies with fat body *hop* depletion, and Yki flies with fat body *Stat92e* depletion at Day 6 (M: Ctrl n=8, Yki n=8, Yki with *hop* depletion n=5, Yki with *Stat92e* depletion n=6; N: Ctrl n=8, Yki n=8, Yki with *hop* depletion n=3, Yki with *Stat92e* depletion n=5;). Statistical significance was assessed by unpaired two-sided Student’s t-test (**b**, **c**, **f**-**i**, **l**) and ordinary one-way ANOVA (**a**, **d**, **e**, **k**, **m**, **n**). ns indicates not significant. Error bars indicate SDs with the mean as the center. Source data

Starvation is a well-known stimulator of gluconeogenesis in animals^62^. To investigate the role of starvation in the increase in gluconeogenesis in Yki flies, we examined the expression levels of *Pepck1* and *Pdk* under conditions of consistent feeding, starvation, and refeeding. Across all three conditions, Yki flies exhibited higher levels of *Pepck1* and *Pdk* expression, suggesting that gluconeogenesis induced in Yki flies and by starvation are additive and operate independently of each other (Fig. 4a,b). At the signalling level, insulin and glucagon are known regulators of gluconeogenesis in mammals^63^. Similarly, insulin and the glucagon-like adipokinetic hormone (Akh) control glucose metabolism in *Drosophila*^64^. To determine whether insulin signalling or Akh regulates gluconeogenesis in the fat body of Yki flies, we depleted the Akh receptor (AkhR) from the fat body. This had no significant effect on trehalose levels (Extended Data Fig. 4a), suggesting that gluconeogenesis in Yki flies is regulated by a different mechanism. Regarding insulin signalling, previous research has indicated that inhibiting *ImpL2* in Yki tumours reduces trehalose levels^43^. Additionally, removing *ImpL2* from Yki tumours led to increased insulin signalling in the fat body, as indicated by a decrease in expression of *InR*, a gene that is upregulated when insulin signalling is low^65^ (Extended Data Fig. 4b). However, *Pepck1* expression levels were not reduced in the fat body of Yki flies with *ImpL2* depletion (Extended Data Fig. 4c). Similarly, depletion of *ImpL2* from the fat body of Yki flies led to decreased *InR* expression but had no significant effect on *Pepck1* levels (Extended Data Fig. 4d,e). As we have previously observed, *ImpL2* is upregulated in different tissues in Yki flies, including gut, fat body, and muscle^43,45^; depletion of *ImpL2* in gut tumours or fat body alone might not be sufficient to affect overall regulation. To thoroughly rule out that *ImpL2* is involved in regulation, we activated insulin signalling in the fat body of Yki flies by overexpressing an active form of InR. Notably, activation of insulin signalling in the fat body did not inhibit the expression of *Pepck1* and *Pdk* in these flies (Fig. 4c,d), further indicating that the induction of gluconeogenesis in Yki flies is independent of insulin signalling.

### The JAK–STAT pathway stimulates fat-body gluconeogenesis

Cachectic factors previously identified in Yki flies include ImpL2 (ref. ^43^), Upd3 (ref. ^44^), and Pvf1 (ref. ^45^). Because ImpL2 does not regulate gluconeogenesis in Yki flies, we turned our attention to Upd3 and Pvf1. We first investigated the Pvf1–PVR–RTK signalling pathway, which has a role in metabolism regulation and has been implicated in inducing bloating in Yki flies^44^. However, activation of the Pvr pathway in wild-type flies did not induce *Pepck1* or *Pdk* expression (Extended Data Fig. 4f–i). Next, we investigated the role of the Upd3–JAK–STAT pathway. Our full-body transcriptome analysis revealed that the fat body is one of the tissues showing prominent activation of JAK–STAT signalling, as evidenced by the upregulation of its target gene, *Socs36E* (Extended Data Fig. 4j). Consistent with this, overexpression of *Upd3* in wild-type fly ISCs (*esg>upd3*) increased fat-body expression of *Pepck1* and *Pdk* (Fig. 4e,f). Notably, two independent chromatin immunoprecipitation followed high-throughput sequencing (ChIP–seq) datasets indicated potential binding sites for the JAK–STAT pathway transcription factor Stat92e in *Pepck1* and *Pdk* (Fig. 4g). To directly assess the regulation of Pepck1 and Pdk by the JAK–STAT pathway, we expressed a tagged, constitutively active form of Stat92e (*Lpp*>*STAT-act-HA*) in the fat body. Consistent with the presence of multiple STAT-binding motifs in these regions, ChIP revealed that Stat92e physically associates with *Pepck1* and *Pdk* in the fat body (Fig. 4h). These observations suggest that JAK–STAT signalling directly promotes fat-body expression of *Pepck1* and *Pdk*, which are essential for the increased gluconeogenesis observed in Yki flies.

Furthermore, we used the dual binary systems to deplete certain genes in the fat body of Yki flies: GAL4–UAS was used to deplete *hop* and *JAK* (*hop*/*JAK*), and LexA–LexAop was used to deplete *Stat92e* (Fig. 4i). Blocking JAK–STAT signalling in the fat body did not affect gut tumours (Fig. 4i–k); however, it significantly inhibited the bloating phenotype in Yki flies (Fig. 4l,m). Notably, depletion of *hop*/*JAK* and *Stat92e* in the fat body of Yki flies led to decreased expression levels of *Pepck1* and *Pdk* (Fig. 4n–q and Extended Data Fig. 4k,l) and resulted in reduced whole-body trehalose levels (Fig. 4r), but did not affect glucose, glycogen, or TAG levels (Fig. 4s and Extended Data Fig. 4m,n). This intervention mirrored the effects observed with the knockdown of either *Pepck1* or *Pdk* in the fat body of Yki flies (Fig. 3 and Extended Data Fig. 3). These findings indicate that inhibition of JAK–STAT signalling in the fat body is sufficient to repress the expression of these gluconeogenic genes and attenuate trehalose levels.

### JAK–STAT inhibition in the fat body rescues cachectic symptoms

**Fig. 5:**
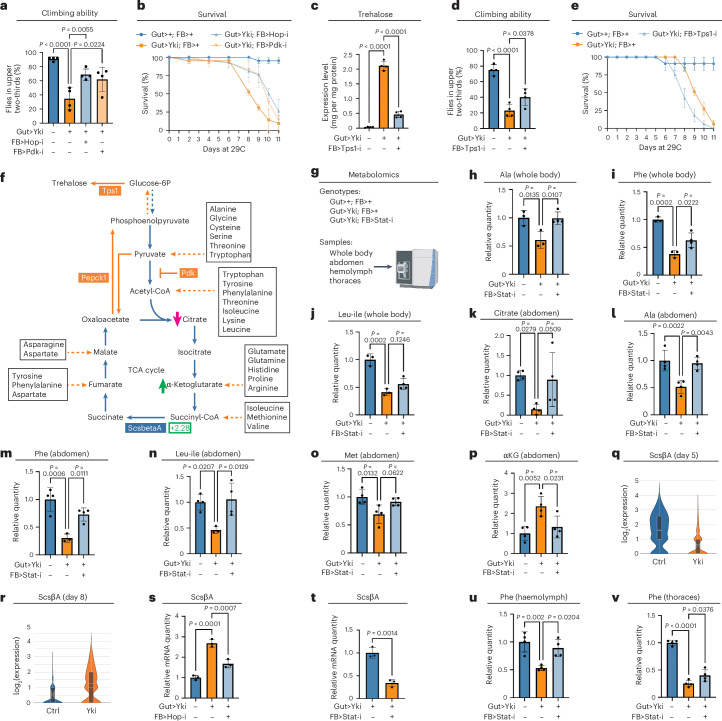
Hepatic gluconeogenesis contributes to cachexia in Yki flies. **a**,**b**, The climbing ability at day 6 (**a**) (*n* = 4) and survival curve (**b**) (*n* = 3) of control flies, Yki flies, and Yki flies with fat-body *hop* or *Pdk* depletion. **c**, Whole-body trehalose levels of control flies, Yki flies, and Yki flies with fat-body *Tps1* depletion at day 8 (*n* = 4). **d**,**e**, The climbing ability at day 7 (**d**) (*n* = 4) and survival curve (**e**) (*n* = 3) of control flies, Yki flies, and Yki flies with fat-body *Tps1* depletion. **f**, An illustration of the metabolic pathway of the TCA cycle and gluconeogenic amino acids. **g**, The experimental design of metabolomics analysis. **h**–**j**, Relative levels of whole-body alanine (**h**), phenylalanine (**i**), and leucine and isoleucine (**j**) (*n* = 4); data were retrieved from metabolomics analysis. **k**–**p**, Relative levels of abdomen citrate (**k**), alanine (**l**), phenylalanine (**m**), leucine and isoleucine (**n**), methionine (**o**), and α-ketoglutarate (**p**) (*n* = 4); data were retrieved from metabolomics analysis. **q**,**r**, Violin plots of expression levels of *ScsβA* at day 5 (**q**) and day 8 (**r**) in the fat-body cell cluster; data were retrieved from snRNA-seq. Box plots denote the median (bottom centre line), mean (top centre line), and interquartile range. The whiskers of each box plot extend to the lowest and highest expression levels. **s**, qRT–PCR analysis of *ScsβA* in the fat body of control flies, Yki flies, and Yki flies with fat body *hop* depletion at day 6 (*n* = 3). **t**, qRT–PCR analysis of *ScsβA* in the fat body of Yki flies and Yki flies with fat body *Stat92e* depletion at day 6 (*n* = 3). **u**, Relative levels of circulating (haemolymph) phenylalanine (*n* = 4); data were retrieved from metabolomics analysis. **v**, Relative levels of thoraces (muscle) phenylalanine (*n* = 4); data were retrieved from metabolomics analysis. Statistical significance was assessed by unpaired two-sided Student’s *t*-test (**t**) and ordinary one-way ANOVA (**a**,**c**,**d**,**h**–**p**,**s**,**u**,**v**). Error bars indicate s.d., with the mean as the centre. See also Extended Data Fig. 5. Source data

**Extended Data Fig. 5:**
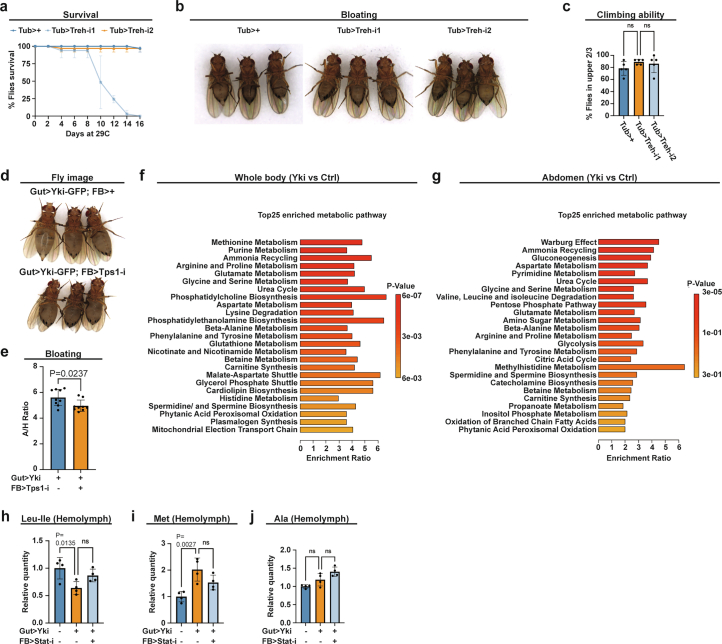
Related to Figure 5. . **(a)** Survival curve of flies with and without whole-body *Treh* depletion (n=3). **(b)** Representative fly image of flies with and without whole-body *Treh* depletion at Day 8. **(c)** Climbing ability of flies with and without whole-body *Treh* depletion at Day 8 (n=5). **(d)** Representative fly image of Yki flies without and with fat body *Tps1* depletion. **(e)** Quantification of bloating phenotypes (abdomen/head size ratio) of Yki flies without and with fat body *Tps1* depletion (n=9). Top 25 enriched metabolic pathways of whole-body **(f)** and abdomen **(g)** metabolites changes between Yki and control flies revealed by metabolomics. Relative levels of hemolymph (circulating) leucine-isoleucine **(h)**, methionine **(i)**, and alanine **(j)** (n=4) revealed by metabolomics. Statistical significance was assessed by unpaired two-sided Student’s t-test (**e**), ordinary one-way ANOVA (**c**, **h**-**j**), and Fisher’s Exact Test (**f**, **g**). ns indicates not significant. Error bars indicate SDs with the mean as the center. Source data

Given that cancer cachexia is a major factor affecting the health and survival^49,66^, we sought to determine whether inhibition of fat-body JAK–STAT signalling in Yki flies could repress cachectic symptoms. We assessed how increased JAK–STAT signalling and hepatic gluconeogenesis in Yki flies influences overall mobility and viability. Inhibition of JAK–STAT signalling (through *hop*/*JAK* depletion) and gluconeogenesis (through *Pdk* depletion) in the fat body of Yki flies both restored climbing ability and improved overall survival (Fig. 5a,b). Considering that Upd3–JAK–STAT signalling drives the expression of *ImpL2* in host tissues, and ImpL2 can suppress trehalase activity^45,67^, we investigated whether the inhibition of trehalase could have a role in inducing bloating. We tested whole-body depletion of trehalase using two *Treh* RNA interference (RNAi) lines (Tub>*Treh* RNAi). Although one line displayed lethality after 8 days, neither line exhibited bloating or impaired climbing ability, indicating that trehalase inhibition does not phenocopy the wasting observed in Yki flies (Extended Data Fig. 5a–c). Altogether, these results suggest that fat-body gluconeogenesis is one of the primary causes of the JAK–STAT-induced cachectic symptoms in Yki flies.

Next, we aimed to determine the pathogenic mechanisms by which the increase in gluconeogenesis contributes to these cachectic phenotypes. A key consequence of activated gluconeogenesis is increased levels of trehalose. In *Drosophila*, trehalose levels are crucial for maintaining body water homeostasis^60^. Therefore, the high levels of trehalose might contribute to the bloating phenotype observed in Yki flies, which could in turn contribute to their climbing defects. To test this, we depleted *Tps1*, which encodes an enzyme that catalyses the final step of gluconeogenesis to generate trehalose, from the fat body (Fig. 2). Consistent with this role, fat-body-specific depletion of *Tps1* significantly reduced trehalose levels and suppressed bloating in Yki flies (Fig. 5c and Extended Data Fig. 5d,e). As expected, this also restored the climbing ability of the flies (Fig. 5d). However, fat-body depletion of *Tps1* did not improve overall survival (Fig. 5e), indicating that high trehalose levels are only a partial cause of the cachectic outcomes observed in Yki flies.

Gluconeogenesis generates trehalose and glucose from non-carbohydrate carbon substrates, such as lactate and glucogenic amino acids^10^ (Fig. 5f). We hypothesized that increased gluconeogenesis causes an imbalance in levels of these substrates. To test this, we used metabolomics to analyse relevant metabolite levels in Yki flies with and without fat-body depletion of *Stat92e*, which we compared with levels in control flies (Fig. 5g). We analysed whole-body samples to assess systemic effects, abdomen samples for local effects of hepatic gluconeogenesis, haemolymph for circulating metabolites, and thoraces as an example of a host organ without tumours that is not undergoing gluconeogenesis (Fig. 5g). Whole-body metabolomics analysis revealed substantial alterations in amino acid metabolism and related metabolic pathways in Yki flies, such as methionine metabolism, ammonia recycling, and arginine and proline metabolism (Extended Data Fig. 5f). Consistent with this finding, we observed reductions in levels of amino acids including alanine, phenylalanine, leucine, and isoleucine, which were either completely or partially restored by depletion of *Stat92e* (Fig. 5h–j). These results indicate that hepatic activation of the JAK–STAT pathway leads to a systemic deficiency in these amino acids.

To further investigate whether the systemic deficiency in these amino acids is due to elevated gluconeogenesis in the fat body, we performed metabolomics analysis on abdomen samples. Consistent with elevated gluconeogenesis in the fat body of Yki flies, we observed an enrichment of metabolites involved in gluconeogenesis and amino acid metabolism, such as valine, leucine, and isoleucine degradation, as well as phenylalanine and tyrosine metabolism (Extended Data Fig. 5g). In line with the observed upregulation of *Pdk*, which blocks pyruvate from entering the tricarboxylic acid (TCA) cycle in Yki flies, we noted significantly decreased levels of citrate, which were restored by *Stat92e* depletion (Fig. 5k). Additionally, several amino acids, including alanine, phenylalanine, leucine, isoleucine, and methionine, were reduced in the abdomen of Yki flies and could be rescued by inhibition of JAK–STAT signalling in the fat body (Fig. 5l–o). These findings suggest that these amino acids are utilized to fuel the TCA cycle in Yki flies (Fig. 5f). Among these amino acids, isoleucine and methionine can be converted to succinyl-CoA, potentially leading to increased levels of α-ketoglutarate (Fig. 5f). Indeed, we observed increased levels of α-ketoglutarate in abdomen samples of Yki flies, along with upregulation of *ScsβA*, a gene required for succinyl-CoA processing (Fig. 5f,p–r). Notably, depletion of *hop*/*JAK* and *Stat92e* in the fat body of Yki flies both decreased *ScsβA* expression levels (Fig. 5s–t), indicating that the changes in metabolites and *ScsβA* expression are linked to the activation of JAK–STAT signalling.

Finally, we examined the effects of fat-body gluconeogenesis on circulating metabolites in the haemolymph. Levels of alanine, leucine, isoleucine, and methionine, which were reduced in the abdomen, did not show the same changes in the haemolymph (Extended Data Fig. 5h–j); however, circulating phenylalanine levels were reduced and could be fully restored by inhibition of JAK–STAT signalling in the fat body (Fig. 5u). Notably, phenylalanine levels were also reduced in the thoraces, which are primarily muscle tissue, and were partially restored by inhibition of JAK–STAT signalling in the fat body (Fig. 5v). These observations indicate that increased levels of gluconeogenesis in the fat body depletes circulating amino acids in the haemolymph, potentially affecting amino acid levels in other host organs. In summary, these data suggest that hepatic activation of JAK–STAT signalling disrupts amino acid homeostasis both locally in the fat body and in other host organs.

### Conserved pathogenic mechanism in mouse models and human

**Fig. 6:**
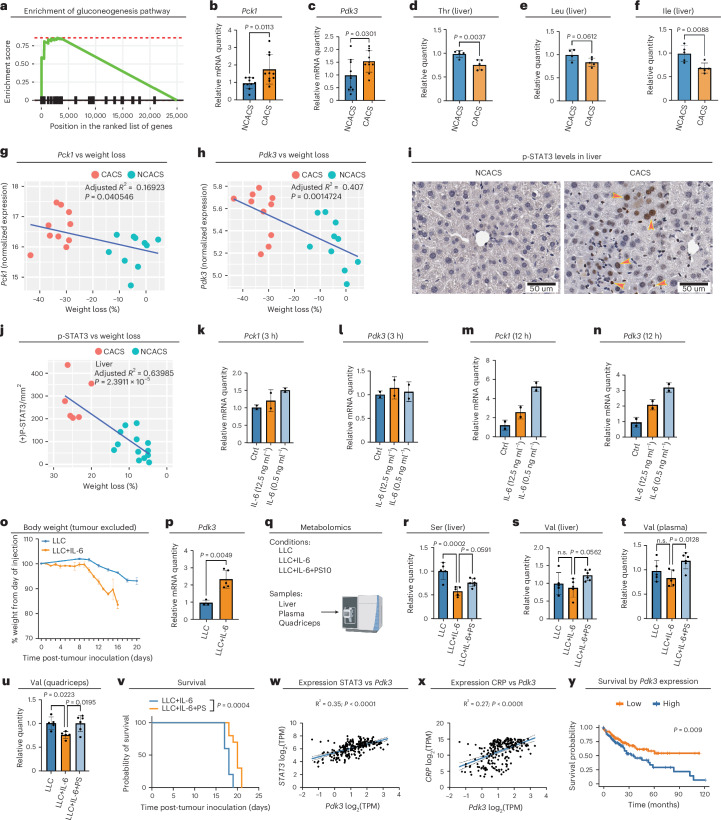
Conserved cachectic role of tumour-induced JAK–STAT signalling. **a**, Gene set enrichment of the gluconeogenesis pathway comparing the livers of CACS to NCACS. **b**,**c**, Expression levels of *Pck1* (**b**) and *Pdk3* (**c**) in livers of NCACS and CACS KL mice; data were retrieved from RNA-seq (*n* = 10). **d**–**f**, Relative levels of liver threonine (**d**), leucine (**e**), and isoleucine (**f**) of NCACS and CACS KL mice (*n* = 5); data were retrieved from metabolomics analysis. **g**,**h**, Correlation plots showing the positive relationship between liver *Pck1* (**g**) and *Pdk3* (**h**) expression (*n* = 10) versus weight loss of KL mice. **i**, IHC staining of p-STAT3 in livers of NCACS and CACS KL mice. **j**, A correlation plot showing the positive relationship between liver p-STAT3 levels (the density of phosphorylated STAT3-positive cells) and weight loss of KL mice (CACS *n* = 4, NCACS *n* = 13). **k**,**l**, qRT–PCR analysis of *Pck1* (**k**) and *Pdk3* (**l**) expression in primary hepatocytes incubated with IL-6 for 3 h (*n* = 2). **m**,**n**, qRT–PCR analysis of *Pck1* (**m**) and *Pdk3* (**n**) expression in primary hepatocytes incubated with IL-6 for 12 h (*n* = 2). **o**, Body weight of B6 mice injected with LLC cells with or without IL-6 expression (*n* = 8). **p**, qRT–PCR of liver *Pdk3* mRNA of B6 mice injected with LLC cells with (*n* = 5) or without (*n* = 3) and IL-6 expression. **q**, The eperimental design of the metabolomics analysis. **r**,**s**, Relative levels of liver serine (**r**) and valine (**s**) of LLC and LLC+IL-6 mice that did or did not receive PS10 (PDK inhibitor) injection (*n* = 5); data were retrieved from metabolomics analysis. **t**, Relative levels of plasma (circulating) valine of LLC and LLC+IL-6 mice that did or did not receive PS10 injection (*n* = 5); data were retrieved from metabolomics analysis. **u**, Relative levels of quadriceps (muscle) valine of LLC and LLC+IL-6 mice that did or did not receive PS10 injection (*n* = 5); data were retrieved from metabolomics analysis. **v**, Survival curve of LLC+IL-6 mice that did or did not receive PS10 injection (*n* = 10). **w**,**x**, Correlation plots showing the positive relationship between expression of *STAT3* and *PDK3* (**w**) and *CRP* and *PDK3* (**x**) in non-diseased liver samples of 226 participants in the GTEx Project. TPM, transcript per million. **y**, Kaplan–Meier survival curves displaying the estimated survival probabilities of people with hepatocellular carcinoma with low (bottom third) and high (top third) hepatic *PDK3* expression; the graph is based on data from the PanCancer Atlas of the TCGA. Statistical significance was assessed by unpaired two-sided Student’s *t*-test (**b**–**f**,**p**), ordinary one-way ANOVA (**r**–**u**), Pearson correlation analysis (**g**,**h**,**j**,**w**,**x**), and log-rank (Mantel–Cox) test (**v**,**y**). Error bars indicate mean and s.d. Panel **q** was created in BioRender (https://BioRender.com/w65y109). See also Extended Data Fig. 6. Source data

**Extended Data Fig. 6:**
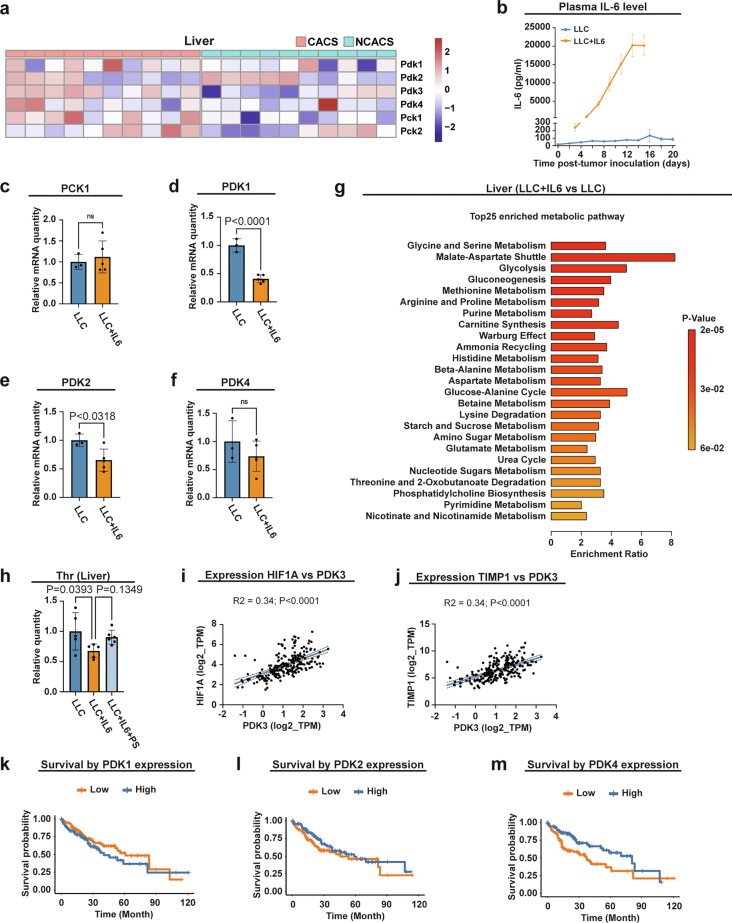
Related to Figure 6. . **(a)** Heatmap plots showing expression levels of *Pdk1-4* and *Pck1-2* in liver of CACS and NCACS KL mice (Columns are showing individual animals, n=10) retrieved by RNA-seq data. **(b)** Plasma IL-6 levels of B6 mice injected with LLC cells without and with IL-6 expression (n=8). qRT-PCR analysis of liver *Pck1* **(c)**, *Pdk1* **(d)**, *Pdk2* **(e)**, and *Pdk4* **(f)** mRNA levels of B6 mice injected with LLC cells without and with IL-6 expression (LLC n=3, LLC+IL6 n=5). **(g)** Top 25 enriched metabolic pathways of liver metabolites changes between B6 mice injected with LLC cells without and with IL-6 expression revealed by metabolomics. **(h)** Relative levels of liver threonine of LLC, LLC+IL-6 mice with and without PS10 (PDK inhibitor) injection (n=5). Correlation plots showing positive correlation between expression of *HIF1A* and *PDK3* **(i)**, *TIMP1* and *PDK3* **(j)** in non-diseased liver samples of 226 individuals from the Genotype-Tissue Expression (GTEx) Project. Kaplan-Meier survival curves displaying the estimated survival probabilities of Hepatocellular Carcinoma patients with low (bottom third) and high (top third) hepatic expression of *PDK1* **(k)**, *PDK2* **(l)**, *PDK4* **(m)**, based on data from the PanCancer Atlas of The Cancer Genome Atlas (TCGA). Statistical significance was assessed by unpaired two-sided Student’s t-test (**c**-**f**), ordinary one-way ANOVA (**h**), and Pearson correlation analysis (**i**, **j**). Error bars indicate SDs with the mean as the center. Source data

To validate these findings in mammals, we used a well-established, inducible, genetically engineered mouse model of lung cancer (*Kras*^*LSL-G12D/+*^;*Lkb1*^*flox/flox*^, referred to as KL mice)^68^. We induced tumours in KL mice through intranasal administration of adenovirus encoding Cre recombinase. Five to six weeks after tumour induction, ~70% of the mice developed cachexia, defined as a total body weight loss of more than 15% (ref. ^68^). To decipher the cachexia-related alterations of glucose metabolism, we used gene set enrichment analysis (GSEA) to compare RNA-seq data from livers of KL mice with cancer anorexia–cachexia syndrome (CACS) with data from KL mice without the condition (NCACS). We found that expression of gluconeogenesis genes was enriched in the livers of the cachectic mice (Fig. 6a). Consistently, *Pck1*, the mouse homologue of fly *Pepck1*, was upregulated in the livers of CACS KL mice (Fig. 6b and Extended Data Fig. 6a). Notably, among the four mouse PDKs (PDK1–PDK4), we observed upregulation of *Pdk3* in the livers of CACS KL mice (Fig. 6c and Extended Data Fig. 6a). PDK3 exhibits the highest catalytic activity among PDKs, is self-activated, and is least sensitive to the inhibition of pyruvate^34,40^; therefore, upregulation of *Pdk3* and *Pck1* in the liver could lead to elevated hepatic production of glucose^11,32,69^. In addition, levels of threonine, leucine, and isoleucine were reduced in the livers of CACS KL mice (Fig. 6d–f), which is reminiscent of what we observed in Yki flies. Of note, liver expression levels of *Pck1* and *Pdk3* positively correlate with the weight loss in KL mice (Fig. 6g,h), suggesting that increased gluconeogenesis contributes to the poor prognosis.

Next, we hypothesized that the cachexia-related expression of *Pck1* and *Pdk3* in CACS KL mice is regulated through the same IL-6–JAK–STAT signalling mechanism. Supporting this hypothesis, we observed elevated levels of phosphorylated STAT3 (p-STAT3) in the livers of CACS KL mice, which were positively correlated with weight loss (Fig. 6I,j). To further confirm this regulation, we treated isolated mouse primary hepatocytes with IL-6. Given the conflicting reports on the effects of IL-6 on gluconeogenesis in primary hepatocytes, particularly regarding varying treatment durations^23,26^, we administered IL-6 for both short (3 h) and long (12 h) periods. As expected, only the longer IL-6 treatment induced the expression of *Pck1* and *Pdk3* (Fig. 6k–n). These results suggest that induction of gluconeogenesis is a chronic pathogenic effect of IL-6.

To investigate the regulation of *Pck1* and *Pdk3* by IL-6 in vivo, we compared B6 mice injected with Lewis lung carcinoma (LLC) cells that were either engineered to express IL-6 or to lack IL-6 expression (Extended Data Fig. 6b). Notably, mice injected with IL-6-producing LLC cells (LLC+IL-6 mice) exhibited rapid weight loss (Fig. 6o). In this context, liver expression of *Pck1* seemed unchanged, probably owing to the absence of necessary coregulators and/or specific conditions (Extended Data Fig. 6c). However, consistent with our observations in KL mice and primary hepatocytes, *Pdk3* was the only PDK isoform upregulated in mice injected with IL-6-producing LLC cells (Fig. 6p and Extended Data Fig. 6d–f). PDK alone can inhibit the synthesis of acetyl-CoA, reducing ATP production through the TCA cycle from glucose^69^, so we used metabolomics to assess the metabolic consequences of *Pdk3* upregulation in this model (Fig. 6q), along with mice injected with PS10, a PDK inhibitor^70^. We observed significant alterations in the gluconeogenesis pathway, as well as amino acid metabolic pathways (Extended Data Fig. 6g). Notably, IL-6 led to a reduction in serine and threonine levels in the liver, which were partially restored by inhibiting PDK3 with PS10 (Fig. 6r and Extended Data Fig. 6h). Notably, PS10 treatment increased valine levels in the liver and plasma, ultimately restoring valine levels in the quadriceps muscle (Fig. 6s–u). These findings suggest that PDK3 alone can impact host amino acid homeostasis, as we observed in Yki flies. Importantly, inhibition of PDK3 significantly improved the survival of LLC+IL-6 mice (Fig. 6v), underscoring the pathogenic role of PDK3 in this mouse model. To assess whether the hepatic *Pdk3* upregulation we observed in the CACS KL and LLC+IL-6 mice is a universal phenomenon or specific to only these models, we analysed liver *Pdk3* expression in other mouse cancer models (Table 1). Indeed, hepatic *Pdk3* upregulation was observed in additional models generated using different tumour-induction methods and in mice with distinct genetic backgrounds. Altogether, these findings suggest that hepatic *Pdk3* induction is a common response to tumours.

**Table 1:** Hepatic *Pdk3* expression in mouse cancer models

Tumour model	Tumour induction	Mouse species	Hepatic *Pdk3* expression	Reference
Colon carcinoma	Implantation of colon carcinoma 26 (C26) cells	CD2F1	Upregulated in cachectic mice	^102^
Breast cancer	Implantation of 4T1 breast cancer cells	BALB/c	Upregulated in cachectic mice	^103^
Paediatric melanoma	Implantation of human-derived melanoma cells MAST360B/SJMEL030083_X2 and MAST552A/SJMEL031086_X3	NCI Ath/nude	Upregulated in cachectic mice	^104^
Melanoma	Implantation of B16F1 melanoma cells	C57BL/6	Upregulated in tumour-bearing mice	^105^
Melanoma	Implantation of B16F10 melanoma cells	C57BL/6	No change	^105^
Breast cancer	Implantation of 67NR breast cancer cells	BALB/c	Upregulated in tumour-bearing mice	^105^
Breast cancer	Implantation of 4T1 breast cancer cells	BALB/c	Upregulated in tumour-bearing mice	^105^
Osteosarcoma	Implantation of K7M2 osteosarcoma cells	BALB/c	Upregulated in tumour-bearing mice	^105^

To extend our findings to humans, we analysed transcriptomic data from non-diseased liver samples of 226 individuals in the Genotype-Tissue Expression (GTEx) Project. IL-6, through STAT3 activation, induces the expression of several target genes, including *CRP*, *HIF1A*, and *TIMP1*^71–74^. We observed a positive correlation between *PDK3* expression and these IL-6–STAT3 pathway targets, as well as the transcription factor gene *STAT3* (Fig. 6w,x and Extended Data Fig. 6i,j), suggesting that IL-6–JAK–STAT signalling regulates *PDK3* expression in humans. To explore the adverse effects of hepatic *PDK3* upregulation, we analysed transcriptomic data from the Hepatocellular Carcinoma cohort of 372 participants in the PanCancer Atlas of The Cancer Genome Atlas (TCGA) Program^75,76^. We assessed the correlation between the expression levels of all four PDK isoforms and survival. In alignment with our mouse model findings, *PDK3* was the only isoform whose elevated expression was significantly associated with reduced survival (Fig. 6y and Extended Data Fig. 6k–m), suggesting that increased hepatic *PDK3* expression contributes to poor outcomes, which is likely to be a result of its role in driving metabolic alterations. Altogether, our results support a conserved mechanism whereby IL-6–JAK–STAT signalling promotes hepatic *PDK3* expression, contributing to mortality linked to cancer.

## Discussion

A major driving force of cancer cachexia is tumour-induced metabolic disorders in host organs^50^. In this study, we leveraged single-nuclei transcriptomics to systemically investigate tumour-induced host metabolism reprogramming and tumour–host organ communication in a *Drosophila* cancer cachexia model. This approach led to the discovery of a previously unknown but conserved cachectic role of Upd3 and IL-6 in induction of hepatic gluconeogenesis and PDK3. Our findings enhance the current understanding of the pathological basis of IL-6 in cancer cachexia, specifically: (1) we captured body-wide gene expression dynamics associated with the progression of cancer cachexia, providing insights into this syndrome; (2) we identified a previously unrecognized regulation of hepatic gluconeogenesis by Upd3–JAK–STAT or IL-6–JAK–STAT signalling, independent of known regulators such as insulin and glucagon; (3) we demonstrated that increased hepatic gluconeogenesis affects host metabolism, both locally in the liver and in other organs, particularly in terms of amino acid homeostasis; (4) we validated our findings in preclinical mouse cancer cachexia models, revealing a previously unknown induction of *Pdk3* by IL-6, thereby filling a knowledge gap regarding the regulation of *Pdk3* in the liver; and (5) we uncovered a previously unknown pathogenic mechanism involving hepatic *Pdk3* expression, suggesting that PDK3 in the liver could be a potential therapeutic target for cancer cachexia.

Our study suggests that reduced anabolism and increased gluconeogenesis underlie the loss of body mass in Yki flies. First, body-mass loss of Yki flies seems to originate from decreased anabolism rather than increased catabolism. We observed decreased lipid and glycogen production in Yki flies at day 8, whereas the corresponding degradation processes were not enhanced. Second, inhibition of hepatic gluconeogenesis alone in Yki flies improved their climbing abilities and survival rates, indicating its contribution to the severity of the cachexia phenotype. Notably, gluconeogenesis is an energy-consuming process that increases overall energy expenditure^14^. Consistent with our findings, elevated energy expenditure is another factor accelerating body wasting in people with cancer^77,78^. Although tumours require energy for rapid cell proliferation, it is unlikely that their metabolic demands significantly contribute to the negative energy balance, given that their energy expenditure is often minor (<5%) when cachexia occurs^79–81^. Therefore, tumour-induced energy expenditure in peripheral organs, such as excessive hepatic gluconeogenesis, could be an underestimated contributor of cachexia^9,14–16,79^.

Notably, in both people with cancer and mouse models, increased hepatic gluconeogenesis does not necessarily lead to hyperglycaemia. For example, in our KL model, CACS mice exhibited lower serum glucose levels. This might be due to glucose consumption by other tissues and the tumour itself, or as a result of cancer-driven malnutrition^1^. These conditions limit the availability of dietary glucose, leading to reduced blood-sugar levels despite increased hepatic gluconeogenesis. Consistent with this, our data indicate that blood-sugar levels partially contribute to the cachexia phenotypes observed in Yki flies, whereas disruptions in amino acid metabolism could play an important role in the observed wasting. These data suggest that altered metabolism in the context of cancer is multifaceted.

Upd3 and IL-6 control hepatic glucose production independent of insulin and glucagon signalling. IL-6 is commonly viewed as a proinflammatory cytokine; however, growing evidence suggests that IL-6 has a broader role in regulating glucose homeostasis^82^. For instance, an early study demonstrated that incubating rat hepatocytes with IL-6 induces gluconeogenesis^23^. Notably, neutralizing IL-6 attenuates elevated hepatic glucose production in high-fat-diet-fed rats, whereas IL-6 infusion promotes hepatic glucose production in control mice^83^. More recently, stress-induced IL-6 has been shown to be essential for the acute hyperglycaemia associated with adaptive ‘fight or flight’ responses^24^. In our study, when their expression was induced by tumours, Upd3 and IL-6 promote gluconeogenesis gene expression through activation of hepatic JAK–STAT signalling in both mouse and fly models. In animals and humans, gluconeogenesis is typically regulated by insulin and glucagon^63,84^. Interestingly, restoration of insulin signalling and inhibition of Akh (glucagon-like hormone) signalling in Yki flies showed no effect on the increased gluconeogenesis, suggesting that JAK–STAT signalling overrides the normal physiological regulation of gluconeogenesis. It is worth noting that IL-6-induced hepatic *Pck1* expression seems to be context-dependent, as evidenced from increased expression of *Pck1* in *Stat3*-knockout mice and STAT3-dependent inhibition of *Pck1* expression in HepG2 cells^26,85,86^. STAT3 exhibited weak binding to the promoter region of *Pck1* (ref. ^86^), suggesting that additional regulators and/or specific conditions, potentially present in certain cancers, could be required for *Pck1* induction. In addition, some studies have reported reduced or unchanged *Pck1* expression in certain cancer cachexia mouse models^87,88^. By contrast, hepatic *Pdk3* upregulation has been consistently observed across several models generated using different tumour-induction methods and in mice with varying genetic backgrounds (Table 1), suggesting a more important role for *Pdk3* in cachexia. Consistent with this, the Upd3–JAK–STAT-dependent induction of *Pdk* expression, or the IL-6–JAK–STAT-dependent induction of *Pdk3* expression, was observed across all tested conditions in both fly and mouse models. Altogether, our study identified a mechanism by which tumours, through Upd3 or IL-6 signalling, induce hepatic gluconeogenesis independently of the Cori cycle, highlighting a direct influence of tumour-derived factors on hepatic glucose metabolism.

Our data suggest that PDK3 could be a potential therapeutic target for cancer cachexia treatment. PDKs play a major role in regulating energy metabolism through controlling the activity of the pyruvate dehydrogenase complex (PDC), which serves as the gatekeeper between glycolysis and the TCA cycle^32,69^. As such, PDKs are closely linked to various physiological energy conditions and numerous human diseases, for instance, starvation and high-fat diets can increase the expression of *Pdk2* and *Pdk4*, as can type 2 diabetes mellitus^89,90^. Among the four PDK isoforms, PDK3 exhibits the highest binding affinity for the inner lipoyl-bearing domain L2 of the transacetylase component E2 of PDC^91^ and the highest phosphorylation rate of pyruvate dehydrogenase at Ser271 (refs. ^92,93^). Moreover, unlike other PDKs, PDK3 is self-activated and resistant to inhibition by pyruvate^34,40^. These observations underscore the distinct physiological role of PDK3. Notably, the regulation of *Pdk3* expression in the liver has remained largely unknown because its expression is almost undetectable under normal conditions, as well as during starvation and diabetes, in both humans and rodents^34,35,39^. Therefore, our discovery that liver *Pdk3* is upregulated in response to IL-6–JAK–STAT signalling highlights a critical and previously unrecognized pathogenic mechanism. Given that PDK3 is highly efficient, autonomously activated and nearly non-repressible^34,40^, its induction in the liver could drive an intense and prolonged inhibition of acetyl-CoA production from pyruvate. This, in turn, could lead to insulin resistance and its associated effects, including increased gluconeogenesis, reduced energy release from the TCA cycle, and decreased fatty acid synthesis. In alignment with this, *PDK3* is the only isoform whose elevated expression was significantly associated with reduced survival in people with hepatocellular carcinoma. Thus, our findings suggest that the IL-6–JAK–STAT-signalling-dependent induction of hepatic *Pdk3* expression represents a previously unknown pathogenic mechanism underlying metabolic disorder in cancer cachexia. Anti-IL-6 antibody treatment in late-stage cachexia has been unsuccessful in clinical trials involving participants with non-small-cell lung cancer, and the pleiotropic effects of IL-6 might lead to side effects such as an increased risk of infections. Identifying a downstream target of IL-6 signalling, such as PDK3, could provide a way to uncouple therapeutic effects from unwanted side effects, potentially offering a more effective treatment for the deregulated metabolic processes observed in cachexia.

In conclusion, we offer insights into the pathogenesis of cancer cachexia using a multi-model approach. Through comprehensive single-cell transcriptome profiling across the entire body in flies, we systematically uncovered the metabolic dysregulation associated with cancer cachexia, identifying the critical role of gluconeogenesis in this process. These findings were further validated in preclinical mouse models and humans. This approach allowed us to reveal the conserved pathogenic role of Upd3–JAK–STAT or IL-6–JAK–STAT signalling in cancer-associated metabolic disorder, highlighting a potential therapeutic strategy of targeting hepatic gluconeogenesis or PDK3 in IL-6-driven cancer cachexia.

### Limitations of the study

We used a fly model to demonstrate the substantial elevation of gluconeogenesis through the hepatic induction of *Pepck1* and *Pdk* expression, and the resulting metabolic aberration. Although *Pdk* and *Pdk3* regulation seems to be conserved between flies and mice, the regulation of *Pepck1* and *Pck1* in mammals might be influenced by additional regulators or conditions that remain unclear at this time. Further studies are needed to identify these potential regulators in mammalian systems. Additionally, this study used two mouse models of lung cancer that are well-established in replicating the clinical progression of the disease from early-stage cancer to cachexia. However, clinical validation of our findings is necessary. We also acknowledge that IL-6 levels in these models differ from those observed in human cancers, which could limit the direct applicability of our findings to clinical practice. The human data in this study are correlative and retrospective, limiting our ability to establish direct causality. However, these analyses provide an important approach for understanding the relationship between IL-6–STAT3 signalling and *PDK3* expression in human liver tissue. Given the ethical and practical constraints of human studies, retrospective data offer valuable insights that complement our mechanistic findings in mouse models. Future research should focus on translating preclinical work such as ours into clinical cancer progression and exploring how these findings could inform the stratified enrollment of participants in interventional trials.

## Methods

### *Drosophila* strains

All flies were kept on standard cornmeal fly food supplemented with yeast and agar. Crosses were grown at 18 °C to inactivate Gal4 and LexA. Adult offspring flies were collected within 48 h after emerging, kept at 18 °C for another 24 h and then incubated at 29 °C for the indicated number of days to induce transgene expression (for example, day 8 indicates that flies were collected after 8 days of transgene expression induction). Flies were given fresh food every 2 days. Stocks used in this study include *esg-Gal4 (P{GawB}NP5130), tub-Gal80ts, UAS-GFP*^43^, *esg-LexA::GAD* (BDSC 66632), *tub-Gal80ts, Lpp-Gal4* (ref. ^94^), *CG31272-Gal4* (BDSC 76171), *UAS-Yki3SA*^95^, *LexAop-Yki3SA-GFP*^61^, *UAS-Pepck1-RNAi* (VDRC 50253), *UAS-Pdk-RNAi* (BDSC 35142), *UAS-Hop-RNAi* (BDSC 32966), *UAS-Stat92e-RNAi* (BDSC 33637), *UAS-ImpL2-RNAi* (NIG 15009R3, BDSC 64936), *UAS-AkhR-RNAi* (BDSC 51710), *UAS-HA-Stat92E* dominant-active form^96^, *UAS-InRca* (BDSC 8263), *UAS-Tps1-RNAi* (BDSC 57488), *UAS-Treh-RNAi* (BDSC 50585, 51810), *UAS-Pvr-act* (BDSC 58496), *UAS-Upd3* (ref. ^97^) and *UAS-Pvf1* (ref. ^98^). *w1118* was used as a control. Female flies were used in all experiments because they had a more notable and consistent bloating phenotype. Genotypes used in this study are described in Supplementary Table 1.

### Whole-body single nuclei profiling of adult flies

Whole-body flies without heads were flash-frozen using liquid nitrogen and were homogenized in 1 ml dounce in buffer consisting of 250 mM sucrose, 10 mM Tris pH 8.0, 25 mM KCl, 5 mM MgCl, 0.1% Triton-X, 0.5% RNasin plus (Promega, N2615), 1× protease inhibitor (Promega, G652A), 0.1 mM DTT, then filtered through 40-µm cell strainer and 40 uµm Flowmi (BelArt, H13680-0040). Samples were centrifuged, washed and resuspended in 1× PBS with 0.5% BSA and 0.5% RNasin plus. The suspension was filtered again with 40 µm Flowmi immediately before FACS sorting. Nuclei were stained with DRAQ7TM Dye (Invitrogen, D15106) and sorted using Sony SH800Z Cell Sorter at Systems Biology Flow Cytometry Facility at Harvard Medical School. After sorting, nuclei were collected and resuspended at 700–800 cells µl^–1^ in 1× PBS buffer with 0.5% BSA and 0.5% RNasin plus.

SnRNA-seq was performed according to the 10X Genomics protocol (Chromium Next GEM Single Cell 3’_v3.1_Rev_D). In brief, 16,000 nuclei were loaded on Chip G for each reaction. Two reactions of control flies and three reactions of Yki tumour flies were processed for each time point (days 5 and 8). Sequencing was conducted using Illumina NovaSeq 6000 S1 at the Harvard Medical School Biopolymers Facility, and reads were aligned to *Drosophila melanogaster* reference genome BDGP6.32. We processed the snRNA-seq data using Cell Ranger count pipeline version 6.1.1 and generated the feature-barcode matrices. The feature-barcode matrices were then processed in R (version 4.3.2) using Seurat (version 5.0.2). Low-quality nuclei with a unique molecular identifier count of <500 were filtered out. In total, 122,818 cells were profiled, including 25,028 control fly cells and 42,404 tumour fly cells at day 5, and 19,046 control fly cells and 36,312 tumour fly cells at day 8. The data were normalized using the NormalizeData function, and 2,000 highly variable features were identified using FindVariableFeatures. The data were scaled, and dimensionality reduction was performed using principal component analysis. Using a graph-based clustering approach, clusters were identified within the data and a UMAP visualization was generated. Marker gene identification was used to annotate 34 unique clusters in the dataset. We visualized the cell clusters and gene expression levels under resolution 0.5 using Loupe Browser 8.

Differential expression analysis was carried out for each cell type using a Wilcoxon rank-sum test between Yki flies and control flies at both days 5 and 8. The cutoffs for differential expression analysis were log_2_(fold change) > 0.38 and *P* < 0.05. GSEA was performed on each cell type using clusterProfiler (version 4.10.1) using the KEGG database. All single-nuclei metrics were generated using ggplot2 (version 3.5.0). Cell–cell communication analysis was performed using FlyPhone. In brief, FlyPhone is a program that calculates cell-type-specific interaction scores between cell types using a permutation test (number of permutations, 1,000) with a curated database of known ligands and receptors. A ligand–receptor interaction with *P* < 0.05 is considered significant. For days 5 and 8, the difference in interaction score was calculated for all significant ligand–receptor pairs in each cell type. Only those with differential expression of the ligand and/or receptor were kept. The number of significantly different ligand–receptor interactions was visualized using a chord diagram from the circlize package (version 0.4.16). Concurrent interactions were calculated as interaction scores, which showed increased or decreased activity in *Yorkie* versus wild type for both day 5 and day 8.

### Metabolite extraction, profiling, and ^13^C tracing in flies

For ^13^C tracing in flies, dissected samples were immediately placed in 1.5-ml Eppendorf tubes on dry ice. After adding 400 µl of ice-cold 80% high-performance liquid chromatography (HPLC)-grade methanol, the samples were homogenized with 0.5 mm beads. Following centrifugation at 20,000*g* for 5 min at 4 °C, the supernatant was transferred to a new Eppendorf tube and vacuum dried. The pellet was resuspended in 20 µl of HPLC-grade water, and 5–7 µl of the resulting solution was injected and analysed using a hybrid 6500 QTRAP triple quadrupole mass spectrometer (AB/SCIEX) connected to a Prominence UFLC HPLC system (Shimadzu). The ^13^C-labelled metabolites were measured, and levels were normalized to the total amount (labelled and unlabelled) of the corresponding metabolite and to [^13^C_3_]alanine in each sample. The normalized values were then indicated as fold changes relative to the control samples. Metabolomics analysis of fly samples was performed following the classic Folch method^99^. In total, 15 adult flies, 40 abdomens, 30 thoraces, and haemolymph from 200–300 adult flies were analysed using lipidomics or metabolomics. Solid samples were homogenized in a 1-ml Dounce homogenizer (Wheaton) on ice with 0.6 ml chloroform and 0.3 ml methanol (2:1 chloroform–methanol mixture). Homogenized samples were transferred to a 15-ml glass tube with a Teflon cap, and an additional 0.6 ml chloroform and 0.3 ml methanol were added to make the final volume approximately 1.8 ml. The haemolymph was transferred to a 15-ml glass tube containing 1.2 ml chloroform and 0.6 ml methanol (2:1 chloroform–methanol mixture). The samples were vortexed briefly and incubated at room temperature on a rotator for 30–45 min. Following incubation, 0.2 volumes (0.36 ml) of HPLC-grade deionized water (HPLC dH_2_O) were added. The mixture was vortexed three times (5 s each), followed by centrifugation at 1,000*g* for 10 min at 4 °C. The upper aqueous phase, containing polar metabolites, was carefully collected using a glass pipette, while avoiding the middle phase. The metabolites were transferred to microcentrifuge tubes, evaporated under vacuum using a SpeedVac rotary evaporator overnight and stored at −80 °C. The polar metabolites were resuspended in 20 μl HPLC-grade water, and then injected and analysed using a hybrid 6500 QTRAP triple quadrupole mass spectrometer (AB/SCIEX) connected to a Prominence UFLC HPLC system (Shimadzu). Analysis was performed using selected reaction monitoring (SRM) to quantify 300 endogenous water-soluble metabolites for steady-state profiling. A subset of metabolites was targeted in both positive- and negative-ion modes, resulting in a total of 311 SRM transitions using polarity switching. The electrospray ionization (ESI) voltage was set to +4,950 V for positive mode and –4,500 V for negative mode. Each SRM transition had a dwell time of 3 ms, with a total cycle time of 1.55 s, acquiring approximately 9–12 data points per detected metabolite. For targeted ^13^C flux analyses, ~140 polar molecules were analysed through 460 SRM transitions. The samples were introduced to the mass spectrometer through hydrophilic interaction chromatography (HILIC) using a 4.6 mm i.d. × 10 cm Amide XBridge column (Waters) with a flow rate of 400 µl min^–1^. The gradient elution program began with 85% buffer B (HPLC-grade acetonitrile) and decreased to 42% B from 0–5 min; 42% B to 0% B from 5–16 min; 0% B was maintained from 16–24 min; followed by a return to 85% B from 24–25 min, with a 7-min hold to re-equilibrate the column. Buffer A consisted of 20 mM ammonium hydroxide and 20 mM ammonium acetate (pH 9.0) in a 95:5 water mixture. Peak areas for each metabolite SRM transition were integrated from the total ion current using MultiQuant v3.2 software (AB/SCIEX). Metabolomics data were analysed using Metaboanalyst 6.0 (https://www.metaboanalyst.ca).

### RT–qPCR of fly samples

For fly samples, the Nucleospin RNA kit (Macherey-Nagel) was used to extract RNA. Complementary DNA was synthesized using the iScript cDNA Synthesis Kit (Bio-Rad, 1708890), according to the manufacturer’s protocol. qPCR was performed with Thermal Cycler CFX 96 Real-Time System qPCR machine using iQ SYBR Green Supermix (Bio-Rad). *RP49* and *CG13220* were used as housekeeping genes. Primers used in qPCR are detailed in the Supplementary Data.

### Protein, lipid, carbohydrate, and body fluid measurements

Protocols for protein, lipid, and carbohydrate measurements were followed, as previously described^43,45^. Four female flies were used for each replicate, and a minimum of three replicates were measured for each sample group. Flies were homogenized in 200 µl 1× PBS with 0.1% Triton X-100 and 1-mm zirconium oxide beads (Next Advance Lab Products, ZROB10) using TissueLyser II homogenizer (Qiagen). Homogenate was incubated at 70 °C for 10 min, and the supernatant was collected after centrifugation at 3,000*g* for 5 min. Five microlitres of supernatant was applied to the Pierce BCA Protein Assay Kit (Thermo Fisher Scientific, 23227) to detect protein levels. TAG and free glycerol levels were quantified from 20 µl supernatant using Triglycerides Reagent (Thermo Fisher Scientific, TR22421) and Free Glycerol Reagent (Sigma-Aldrich, F6428), respectively. Free glycerol was subtracted from TAG values. Glucose levels were measured from 10 µl supernatant using Infinity Glucose Hexokinase Reagent (Thermo Fisher Scientific, TR15421) or the d-Glucose Assay Kit (Megazyme, K-GLUC). Glycogen levels were measured in the same manner as glucose levels, but supernatant was incubated with 1/500 amyloglucosidase (Sigma-Aldrich, A1602). Trehalose levels were measured in the same manner as glycogen levels, but supernatants were incubated with trehalase (Megazyme, E-TREH). The amount of glucose was subtracted from trehalose and glycogen read values. TAG, free glycerol, glucose, and trehalose levels were normalized to corresponding protein levels in each sample. Body fluids were assessed as described previously^47^; in brief, weighed flies were dried at 65 °C for 5 h and then weighed again. The quantity of body fluid was calculated by subtracting the second measurement from the first.

### Climbing index and survival curve of flies

To assess the climbing ability, flies were transferred to a new vial and then tapped down to the bottom. Vials were imaged after 4 s. Percentages of flies in the upper two-thirds of the vial were recorded. Four independent vials for each genotype were tested to generate the climbing index. Survival of flies was analysed by calculating the percentage of flies alive in each vial incubated at 29 °C. Three vials of flies from each genotype were tested, and flies were placed in vials with fresh food daily.

### Gut and fly imaging

Adult fly guts were dissected in cold 1× PBS and fixed for 30 min in 1× PBS with 4% formaldehyde. Samples were washed three times in 1× PBS with 0.3% Triton X-100 (PBT) and then mounted in Vectashield with DAPI (Vector Laboratories, H-1200). For pH3 staining, washed guts were incubated with blocking buffer (5% NDS in PBT) for 1 h at room temperature. Rabbit anti-pH3 (1:1,000, Millipore, 06-570) primary antibodies were incubated overnight at 4 °C. Gut was washed three times in PBT before incubation with the secondary antibody, donkey anti-rabbit 565 (1:2,000, Molecular Probes, A31572), for 1 h. After three washes in PBT, guts were mounted in Vectashield with DAPI (Vector Laboratories, H-1200). Confocal images were captured with the Nikon Ti and Ti2 Spinning Disk at the Microscopy Resources on the North Quad (MicRoN) core facility at Harvard Medical School, using Nikon Elements Acquisition Software AR (v5.02). Tumours were quantified by measuring the average GFP signalling strength of gut tumours from individual flies using Fiji-imageJ. Adult fly phenotypes were captured using a ZEISS Axiozoom V16 fluorescence microscope. Bloating was quantified as the ratio between abdomen and head compartment size using Fiji-imageJ.

### Chromatin immunoprecipitation

The ChIP assay was performed using the SimpleChIP Plus Enzymatic Chromatin IP Kit (Cell Signaling, 9005). For each immunoprecipitation, fat bodies from 50 adult flies with HA-tagged dominant-active Stat92e fat body expression were dissected and flash-frozen in liquid nitrogen. Samples were cross-linked with 1.5% formaldehyde for 20 min at room temperature. After cross-linking was stopped by the addition of glycine solution for 5 min at room temperature, samples were washed twice with 1 ml 1× PBS containing 1× Protease Inhibitor Cocktail (PIC) and disaggregated using a 1-ml Dounce homogenizer. Nuclei were prepared according to the manufacturer’s protocol and were lysed using Diagenode Bioruptor sonicator to release the cross-linked chromatin. Chromatin was diluted in 1× ChIP buffer and incubated with 10 µl HA-tagged (C29F4) rabbit monoclonal antibodies (Cell Signaling, 3724) or normal rabbit IgG (Cell Signaling, 2729) overnight at 4 °C with rotation. Thirty microlitres of ChIP-Grade Protein G Magnetic Beads (Cell Signaling, 9006) was incubated with each immunoprecipitation for 2 h at 4 °C with rotation. Beads were washed and incubated in 150 µl 1× ChIP Elution Buffer at 65 °C for 30 min with vortexing (1,200 r.p.m.) to elute the chromatin. Cross-links were reversed by adding 6 µl 5 M NaCl and 2 µl Proteinase K to the eluted chromatin supernatant and incubating 2 h at 65 °C. DNA was purified from each sample using Spin Columns provided by the kit. 1 ul DNA sample was used as template for qPCR to detect enrichments of certain DNA regions. qPCR of a fragment in the Sam-S gene region was used as the negative control. Primers used for ChIP–qPCR are detailed in the Supplementary Data.

### Mouse models

The *Kras*^*G12D*/+^;*Lkb1*^*f/f*^ mice have been described before^100^; these mice were further backcrossed to FVB mice. The mice were kept on a 24-h 12 h–12 h light–dark cycle at 22 °C and received rodent chow from PicoLab (Rodent 20, 5053; Lab Diet, 3.43 kcal g^–1^) and free access to water. Tumour induction in adult FVB mice (12–20 weeks old) was achieved by intranasal administration of 75 μl PBS containing 2.5 × 10^7^ pfu of Adenovirus CMV-Cre (Ad5CMV-Cre) obtained from the University of Iowa Gene Transfer Vector Core and 1 mM CaCl_2_. We had previously defined mice as CACS if they lost more than 15% of their body weight from their peak weight over the course of the experiment, otherwise they were classified as NCACS^68^. Male mice were used for liver RNA-seq, both male and female mice were used in all other experiments. The *Kras*^*G12D*/+^;*Lkb1*^*f/f*^ mice experiments were approved by the Institutional Animal Care and Use Committee (IACUC) at the Weill Cornell Medical College and were maintained as approved by the IACUC at Weill Cornell Medicine. In KL mice, tumours develop in the lungs and cannot be directly measured. Therefore, instead of assessing tumour size or mass, we monitored overall mouse health as an indicator of disease progression. Tumour-bearing KL mice were weighed, inspected, and monitored at least 3 times per week for signs of pain, distress, or clinical deterioration. If mice reached a body-condition score of 2, or appeared distressed, lethargic, hunched, or showed more than 25% weight loss, they were humanely euthanized using CO_2_. C57BL/6J mice were obtained from the Jackson Laboratory (strain no. 000664). After a week of acclimation, 2 ×10^6^ LLC cells or LLC cells edited to produce IL-6 (LLC+IL-6) were subcutaneously inoculated into their right flank. Mice were kept in pathogen-free conditions on a 24-h 12 h–12 h light–dark cycle at 23 °C. Mice were fed a PicoLab Rodent Diet 20 LabDiet (5053) ad libitum. Eight-week-old male C57BL/6J mice were used in the experiments. For PDK inhibition treatment, PS10 (MedChemExpress, HY-121744) was administered intraperitoneally at a dosage of 70 mg kg^–1^ per day to mice weighing approximately 23 g. C57BL/6J mice experiments were approved by the IACUC at Cold Spring Harbor Laboratory and were conducted in accordance with the National Institutes of Health Guide for the Care and Use of Laboratory Animals. The maximum tumour size permitted was 2 cm on any axis. This maximum tumour size or burden was not exceeded. Body weights and clinical signs of cachexia were monitored on a daily basis. Handling was kept to a minimum. Mice were euthanized when their tumour size exceeded 2 cm length, when weight loss exceeded 15% of peak weight, or when they showed clinical signs of discomfort indicative of a cachectic endpoint, as assessed by the Animal Cachexia Score (ACASCO), including piloerection, diarrhea or constipation, hunched posture, tremors and closed eyes. Death was confirmed by cervical dislocation^101^. Mice injected with LLC+IL-6 cells reached the >15% body weight loss endpoint. Mice in the LLC group were euthanized 22–24 days after injection of the LLC cell line, when tumours reached 2 cm in length. LLC group mice did not reach the cachectic endpoint but did exhibit a mild cachectic phenotype, characterized by reduced adipose and muscle tissue mass, and splenomegaly compared with non-tumour-bearing control group mice.

### Plasma measurements from C57BL/6J mice

Tail vein bleeds were performed using a scalpel for tail venesection. Mice were not restrained. Plasma samples were collected using heparin-coated haematocrit capillary tubes to prevent coagulation and were processed as follows: centrifuged at 2,000*g* for 30 min at 4 °C, snap frozen in liquid nitrogen and stored at −80 °C. IL-6 levels were measured from plasma using the mouse IL-6 Quantikine ELISA Kit (R&D Systems, M6000B).

### Lewis lung carcinoma cell line

LLC cells were purchased from American Type Culture Collection (ATCC) (LL/2-LLC1; CRL-1642) and cultured in complete growth medium consisting of Dulbecco’s Modified Eagle Medium (DMEM) (Corning, 10027CV) containing 10% of heat-inactivated fetal bovine serum (FBS) (Thermo Fisher Scientific, 10-438-026) and 1× penicillin–streptomycin solution (Thermo Fisher Scientific, 15-140-122) under sterile conditions. 1x Trypsin-EDTA (Thermo Fisher Scientific, 15400054) was used for cell dissociation. Cells were resuspended in FBS-free DMEM, and viable cells were counted using a Vi-Cell counter prior to subcutaneous injection of 2 × 10^6^ viable cells diluted in 100 μl DMEM into the right flank of each C57BL/6J mouse.

LLC cells were edited to constitutively produce IL-6. LLC cells were seeded into 24-well plates with 50,000 cells per well. After 24 h, they were transfected with a total of 500 ng of plasmid (comprising 2.5:1 PB-IL6 plasmid and PBase plasmids) using Lipofectamine 3000 (Thermo Fisher), according to the manufacturer’s protocol. PBase plasmid was obtained from System Biosciences (PB210PA-1), and PB-IL6, comprising mouse Il6 cDNA driven by EF1a promoter and flanked by piggyBac elements, was obtained from VectorBuilding. After 48 h, the medium was changed and replaced with DMEM supplemented with 3 μg ml^–1^ puromycin. After 14 days of antibiotic selection, the medium was replaced with DMEM for 24 h, followed by isolation of monoclonal populations by serial dilutions in a 96-well plate. To identify clones with constitutive IL-6 expression, we measured IL-6 in the cell supernatant for each clone using the Mouse IL-6 ELISA Kit (Abcam, ab222503).

### Metabolites extraction and profiling in mice

Metabolite extract from mouse samples were analysed using a quadrupole-orbitrap mass spectrometer coupled with hydrophilic interaction chromatography (HILIC) as the chromatographic technique. Chromatographic separation was achieved on an XBridge BEH Amide XP Column (2.5 µm, 2.1 mm × 150 mm) with a guard column (2.5 µm, 2.1 mm × 5 mm) (Waters). For the gradient, mobile phase A was composed of a 95:5 ratio of water to acetonitrile, and mobile phase B was composed of water:acetonitrile 20:80; both phases contained 10 mM ammonium acetate and 10 mM ammonium hydroxide. The linear elution gradient was: 0–3 min, 100% B; 3.2–6.2 min, 90% B/10% A; 6.5–10.5 min, 80% B/20% A; 10.7–13.5 min, 70% B/30% A; 13.7–16 min, 45% B/55% A; and 16.5–22 min, 100% B, with a flow rate of 0.3 ml min^–1^. The autosampler was kept at 4 °C. The injection volume was 5 µl. Needle wash was applied between samples using a 40:40:20 ratio of methanol to acetonitrile to water. The Orbitrap Exploris 480 (Thermo Fisher Scientific) mass spectrometer was used, scanning from 70 to 1,000 *m/z* in alternating polarity. The resolution was ×120,000. Metabolites were identified on the basis of accurate mass and retention time using the EI-Maven (Elucidata) with an in-house library.

### Bulk RNA sequencing from livers of KL mice

Total RNA was extracted from the livers using TRIzol (Thermo Fisher Scientific), followed by a clean-up step using the RNeasy kit (Qiagen). One microgram of total RNA from each sample was submitted to the WCM Genomics Resources Core Facility. Raw sequenced reads were aligned to the mouse reference GRCm38 using STAR (v2.4.1d, 2-pass mode) aligner, and raw counts were obtained using HTSeq (v0.6.1). Differential expression analysis, batch correction, and principal component analysis were performed using R Studio Version 4.2.2 and DESeq2 (v.1.38.3). GSEA analysis was performed with the R package fGSEA (10.18129/B9.bioc.fgsea), using the Reactome pathway database contained in the 2022 release of Mouse Molecular Signatures Database from the Broad Institute (https://www.gsea-msigdb.org/gsea/msigdb/mouse/collections.jsp).

### Immunohistochemistry

Mouse tissues were fixed overnight in 4% paraformaldehyde (PFA) and stored in 70% ethanol. Subsequent paraffin embedding and sectioning at 5-μm thickness were performed at Histowiz. For immunohistochemistry (IHC) of STAT3 phosphorylated at Tyr705, slides were deparaffinized using Histoclear (National Diagnostics), rehydrated, and subjected to antigen retrieval in a pressure cooker for 13 min using Tris-EDTA buffer (10 mM Tris base, one mM EDTA, 0.05% Tween 20, pH 9.0). Endogenous peroxidase activity was quenched with 3% hydrogen peroxide in PBS for 5 min, and non-specific binding was blocked using 5% donkey serum. The primary antibody rabbit anti-phospho-Stat3 (Tyr705) (D3A7) (Cell Signaling Technology, 9145, 1:100) was applied to the slides and incubated overnight. Then, secondary antibodies (ready-to-use biotinylated goat anti-rabbit-IgG antibody (H+L); Vector, BP-9100-50), avidin–biotin complex reagents (PK-4100) and DAB peroxidase substrate from Vector Laboratories (SK-4100) were applied. Imaging was done using a Zeiss Axioscope Imager at Histowiz. IHC quantifications were automated using whole-slide scans and QuPath software (0.4.2).

### Primary hepatocyte isolation

Male mice (8- to 10-week-old C57BL/6 mice) were used for primary hepatocyte isolation. Ketamine (100 mg kg^–1^ body weight) and xylazine (10 mg kg^–1^ body weight), were used for anaesthesia. After laparotomy, suprahepatic veins were clamped through an incision in the diaphragm followed by liver perfusion through the inferior vena cava with 10 ml of warm liver perfusion medium (Gibco, 17701-038) and 35 ml of warm liver digest medium (Gibco, 7703-034). After 6–10 min of perfusion, the liver was dissected, placed in ice-cold hepatocyte wash medium (Gibco, 17704-024). To release hepatocytes into the medium, the Glisson capsule was gently peeled off. The cell suspension was then filtered through a 70-µm cell strainer (Falcon, 352350), and the cells were washed once (80*g*, 5 min, 4 °C). The hepatocytes were further purified using a Percoll solution (18 ml Percoll, Sigma, P1644-500ML; 2 ml of Hanks base 10×, Sigma, H4641-100ML), centrifuged for 10 min at 150*g* and 4 °C, and then washed and resuspended in Williams E medium (Life Technologies, 12551032) with 10% FBS. The cells were then plated on six-well collagen-coated Primaria plates (Corning) at a density of 5 × 10^5^ per well. After 4 h, the cells were washed with warm PBS and replaced with Williams E medium containing no FBS for 16 h, when the experiments were performed.

### RT–qPCR of mouse samples

For LLC and IL-6-secreting LLC mouse samples, 100 mg of liver tissue was lysed in 1 ml of QIAzol using the Qiagen TissueLyser II at 20 Hz for 4 min. Samples were centrifuged at 12,000*g* at 4 °C for 12 min to separate the aqueous layer from the organic layer and any debris. Approximately 600 µl of RNA containing the aqueous layer was collected and processed using Qiagen RNeasy lipid RNA extraction kit and QiaCube machine to isolate RNA in 50-µl elution volumes. RNA concentrations of all samples were quantified, and samples were further diluted using ddH_2_O to reach a standard concentration of 100 ng µl^–1^ for each sample. TaqMan RNA-to-Ct 1 step kit was used for qPCR following the provided protocol for 10-ml reaction volumes. Data were analysed using the 2^–∆∆^^CT^ method. *Gapdh* and *Ppia* were used as housekeeping genes. The primers used were *Pck1* (Thermo Fisher Scientific, Mm01247058_m1), *Pdk2* (Thermo Fisher Scientific, Mm00446681_m1), *Pdk3* (Thermo Fisher Scientific, Mm00455220_m1), *Igfbp3* (Thermo Fisher Scientific, cMm01187817_m1), *Gapdh* (Thermo Fisher Scientific, Mm99999915_g1), and *Ppia* (Thermo Fisher Scientific, Mm02342430_g1).

### Public datasets, software, quantification, and statistical analyses

Transcriptomic data from non-diseased liver samples of 226 individuals were obtained from the GTEx Project. Additionally, transcriptomic data and corresponding survival records were obtained from the Hepatocellular Carcinoma (LIHC) cohort of 372 participants in the PanCancer Atlas of TCGA Program^75,76^. Sex and gender were not considered in the design of present research, and related information was not collected for the analysis. Kaplan–Meier survival curves were generated using the R packages survival and survminer. We used Biorender to generate a subset of figures, and ChatGPT to proofread parts of the text. After using ChatGPT, the authors reviewed and edited the content as needed and take full responsibility for the content of the publication. GraphPad Prism was used for statistical analysis, gene expression correlation analysis, and generation of figures. Data distribution was assumed to be normal, but this was not formally tested. Animals and samples were randomly assigned to experimental groups, and the data collection was randomized. Experimenters doing data collection and analysis were not blinded to the conditions of the experiments. No animals or data points were excluded from the analyses. No statistical methods were used to pre-determine sample sizes but our sample sizes are similar to those reported in previous publications^44,45,68^. Statistical analysis was done with Student’s *t*-test (two experimental groups) or ordinary one-way ANOVA (three or more experimental groups) by the default settings of GraphPad Prism. Gene expression levels (assessed through qPCR) were normalized to the mean of control samples. *n* indicates the number of biological replicates in each experiment. Error bars indicate the s.d., with the mean as the centre.

### Reporting summary

Further information on research design is available in the Nature Portfolio Reporting Summary linked to this article.

## Supplementary information

Supplementary Information Supplementary Figure 1 and Supplementary Table 1

Reporting Summary

Supplementary Data Primers used in this study

## Source data

Source Data Fig. 3 Source Data for all data presented in graphs within figure 3

Source Data Fig. 4 Source Data for all data presented in graphs within figure 4

Source Data Fig. 5 Source Data for all data presented in graphs within figure 5

Source Data Fig. 6 Source Data for all data presented in graphs within figure 6

Source Data Extended Data Fig. /Table 3 Source Data for all data presented in graphs within Extended Data figure 3

Source Data Extended Data Fig. /Table 4 Source Data for all data presented in graphs within Extended Data figure 4

Source Data Extended Data Fig. /Table 5 Source Data for all data presented in graphs within Extended Data figure 5

Source Data Extended Data Fig. /Table 6 Source Data for all data presented in graphs within Extended Data figure 6

## Data Availability

*Drosophila* Raw snRNA-seq reads have been deposited in the NCBI Gene Expression Omnibus (GEO) database under accession code GSE229526. Processed datasets can be mined through a web tool (https://www.flyrnai.org/scRNA/body/) that allows users to explore genes and cell types of interest. The raw RNA-seq data for ten liver samples from KL mice are available in the GEO database under the accession code GSE107470. An additional ten samples, specifically processed for this manuscript, can be accessed under the accession number GSE286259. Liver transcriptomic data for non-diseased people and people with cancer are publicly available from Genotype-Tissue Expression (GTEx) Project and PanCancer Atlas of The Cancer Genome Atlas (TCGA) Program, respectively. Other data that support the plots within this paper and other findings of this study are provided as source data files. Source data are provided with this paper.
