# Supplementary material for: Hepatic gluconeogenesis and PDK3 upregulation drive cancer cachexia in flies and mice
Source: Nat Metab. 2025 Apr 16;7(4):823–41. doi: 10.1038/s42255-025-01265-2 (PMC12021660; doi:10.1038/s42255-025-01265-2)
Supplement: Supplementary file 1 — Supplementary Figure 1 and Supplementary Table 1 [file 42255_2025_1265_MOESM1_ESM.pdf]

nature metabolism

Supplementary information

https://doi.org/10.1038/s42255-025-01265-2

Hepatic gluconeogenesis and PDK3 upregulation drive cancer cachexia in flies and mice

In the format provided by the authors and unedited

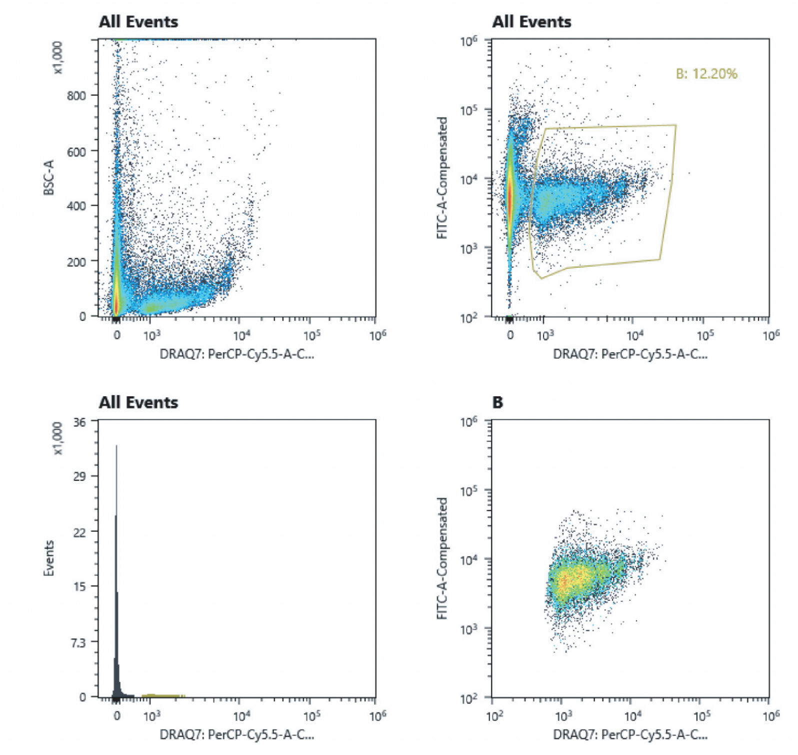

Supplementary figure 1. Gating strategy for the flow cytometry

Flow cytometric sorting of nuclei from fly full-bod samples. Nuclei were stained with fluorescent DNA dye DRAQ7, multiple nuclei populations indicate polyploid cells.

Supplementary table 1. Genotypes used in this study

Figure	
1&S1<br>2&S2<br>3B-H&S3A-C, J-K<br>S4J<br>5Q-R	*esg-GAL4, tub-GAL80TS > UAS-GFP*<br>*esg-GAL4, tub-GAL80TS > UAS-GFP, UAS-yki*^{3SA}
S3D	*esg-GAL4, tub-GAL80TS > UAS-GFP, UAS-yki*^{3SA}<br>*esg- LexA, tub-GAL80TS > LexAop-yki*^{3SA}*-GFP 2nd*; *Lpp>+*<br>*esg- LexA, tub-GAL80TS > LexAop-yki*^{3SA}*-GFP 3rd*; *Lpp>+*
3I-U<br>S3E-I	*esg-LexA, tub-GAL80TS > +*; *Lpp>+*<br>*esg- LexA, tub-GAL80TS > LexAop-yki*^{3SA}*-GFP 2nd*; *Lpp>+*<br>*esg- LexA, tub-GAL80TS > LexAop-yki*^{3SA}*-GFP 2nd*; *Lpp>Pepck-i*<br>*esg- LexA, tub-GAL80TS > LexAop-yki*^{3SA}*-GFP 2nd*; *Lpp>Pdk-i*
4A-B	*esg-LexA, tub-GAL80TS > +*; *Lpp>+*<br>*esg- LexA, tub-GAL80TS > LexAop-yki*^{3SA}*-GFP 2nd*; *Lpp>+*
4C-D	*esg-LexA, tub-GAL80TS > +*; *Lpp>+*<br>*esg- LexA, tub-GAL80TS > LexAop-yki*^{3SA}*-GFP 3nd*; *Lpp>+*<br>*esg- LexA, tub-GAL80TS > LexAop-yki*^{3SA}*-GFP 3nd*; *Lpp>InRca*
4E-F	*esg-GAL4, tub-GAL80TS > UAS-GFP*<br>*esg-GAL4, tub-GAL80TS > UAS-GFP, UAS-Upd3*
4H	*Lpp-GAL4, tub-GAL80TS > +*<br>*Lpp-GAL4, tub-GAL80TS > UAS-STAT-act*
4I-S<br>S4K-N<br>5S-T	*esg-LexA, tub-GAL80TS > +*; *Lpp>+*<br>*esg- LexA, tub-GAL80TS > LexAop-yki*^{3SA}*-GFP 2nd*; *Lpp>+*<br>*esg- LexA, tub-GAL80TS > LexAop-yki*^{3SA}*-GFP 2nd*; *Lpp>Hop-i*<br>*esg- LexA, tub-GAL80TS > LexAop-yki*^{3SA}*-GFP 2nd*; *Lpp>Stat92e-i*
S4A	*esg-LexA, tub-GAL80TS > +*; *Lpp>+*<br>*esg- LexA, tub-GAL80TS > LexAop-yki*^{3SA}*-GFP 3rd*; *Lpp>+*<br>*esg- LexA, tub-GAL80TS > LexAop-yki*^{3SA}*-GFP 3rd*; *Lpp>AkhR-i*
S4B-C	*esg-GAL4, tub-GAL80TS > UAS-GFP, UAS-yki*^{3SA}

	esg-GAL4, tub-GAL80TS > UAS-GFP, UAS-yki^{3SA}*, UAS-ImpL2-i*
S4D-E	*esg-LexA, tub-GAL80TS > +*; *Lpp>+*<br>*esg- LexA, tub-GAL80TS > LexAop-*yki^{3SA}*-GFP 2nd*; *Lpp>+*<br>*esg- LexA, tub-GAL80TS > LexAop-*yki^{3SA}*-GFP 2nd*; *Lpp>ImpL2-i*
S4F-G	*esg-GAL4, tub-GAL80TS > UAS-GFP*<br>*esg-GAL4, tub-GAL80TS > UAS-GFP, UAS-Pvf1*
S4H-I	*Lpp-GAL4, tub-GAL80TS > W1118*<br>*Lpp-GAL4, tub-GAL80TS > PvRact*
5A-B	*esg-LexA, tub-GAL80TS > +*; *Lpp>+*<br>*esg- LexA, tub-GAL80TS > LexAop-*yki^{3SA}*-GFP 2nd*; *Lpp>+*<br>*esg- LexA, tub-GAL80TS > LexAop-*yki^{3SA}*-GFP 2nd*; *Lpp>Hop-i*<br>*esg- LexA, tub-GAL80TS > LexAop-*yki^{3SA}*-GFP 2nd*; *Lpp>Pdk-i*
S5A-C	*Tub-GAL4, tub-GAL80TS > W1118*<br>*Tub-GAL4, tub-GAL80TS > Treh-i1*<br>*Tub-GAL4, tub-GAL80TS > Treh-i2*
5C-E<br>S5D-E	*esg-LexA, tub-GAL80TS > +*; *Lpp>+*<br>*esg- LexA, tub-GAL80TS > LexAop-*yki^{3SA}*-GFP 3rd*; *Lpp>+*<br>*esg- LexA, tub-GAL80TS > LexAop-*yki^{3SA}*-GFP 3rd*; *Lpp>Tps1-i*
5H-P&U-V<br>S5F-J	*esg-LexA, tub-GAL80TS > +*; *Lpp>+*<br>*esg- LexA, tub-GAL80TS > LexAop-*yki^{3SA}*-GFP 2nd*; *Lpp>+*<br>*esg- LexA, tub-GAL80TS > LexAop-*yki^{3SA}*-GFP 2nd*; *Lpp>Stat92e-i*
6A-J<br>S6A	$Kras^{LSL\text{-}G12D/+}$; $Lkb1^{flox/flox}$
6O-V<br>S6B-H	*C57BL/6J LLC*<br>*C57BL/6J LLC + IL-6*
