# Supplementary material for: Hepatic gluconeogenesis and PDK3 upregulation drive cancer cachexia in flies and mice
Source: Nat Metab. 2025 Apr 16;7(4):823–41. doi: 10.1038/s42255-025-01265-2 (PMC12021660; doi:10.1038/s42255-025-01265-2)
Supplement: Supplementary file 2 — Reporting Summary [file 42255_2025_1265_MOESM2_ESM.pdf]

nature portfolio

Corresponding author(s): Ying Liu and Norbert Perrimon

Last updated by author(s): Feb 19, 2025

Reporting Summary

Nature Portfolio wishes to improve the reproducibility of the work that we publish. This form provides structure for consistency and transparency in reporting. For further information on Nature Portfolio policies, see our Editorial Policies and the Editorial Policy Checklist.

Statistics

For all statistical analyses, confirm that the following items are present in the figure legend, table legend, main text, or Methods section.

n/a	Confirmed	
☐	☒	The exact sample size (*n*) for each experimental group/condition, given as a discrete number and unit of measurement
☐	☒	A statement on whether measurements were taken from distinct samples or whether the same sample was measured repeatedly
☐	☒	The statistical test(s) used AND whether they are one- or two-sided<br>*Only common tests should be described solely by name; describe more complex techniques in the Methods section.*
☒	☐	A description of all covariates tested
☒	☐	A description of any assumptions or corrections, such as tests of normality and adjustment for multiple comparisons
☐	☒	A full description of the statistical parameters including central tendency (e.g. means) or other basic estimates (e.g. regression coefficient) AND variation (e.g. standard deviation) or associated estimates of uncertainty (e.g. confidence intervals)
☐	☒	For null hypothesis testing, the test statistic (e.g. *F*, *t*, *r*) with confidence intervals, effect sizes, degrees of freedom and *P* value noted<br>*Give P values as exact values whenever suitable.*
☒	☐	For Bayesian analysis, information on the choice of priors and Markov chain Monte Carlo settings
☒	☐	For hierarchical and complex designs, identification of the appropriate level for tests and full reporting of outcomes
☒	☐	Estimates of effect sizes (e.g. Cohen's *d*, Pearson's *r*), indicating how they were calculated

Our web collection on statistics for biologists contains articles on many of the points above.

Software and code

Policy information about availability of computer code

Data collection	Nikon Elements Acquisition Software AR (v5.02)
Data analysis	Cellranger (6.1.1), Loupe Browser(8.0), R Studio (4.2.2) , DESeq2 (,v.1.38.3) FlyPhone (onine tool), GraphPad Prism (10), Fiji ImageJ (2.16.0), Seurat (5.0.2)

For manuscripts utilizing custom algorithms or software that are central to the research but not yet described in published literature, software must be made available to editors and reviewers. We strongly encourage code deposition in a community repository (e.g. GitHub). See the Nature Portfolio guidelines for submitting code & software for further information.

Data

Policy information about availability of data

All manuscripts must include a data availability statement. This statement should provide the following information, where applicable:

- Accession codes, unique identifiers, or web links for publicly available datasets
- A description of any restrictions on data availability
- For clinical datasets or third party data, please ensure that the statement adheres to our policy

Drosophila Raw snRNA-seq reads have been deposited in the NCBI Gene Expression Omnibus (GEO) database under accession codes: GSE229526. Processed datasets can be mined through a web-tool [https://www.flyrnai.org/scRNA/body/] that allows users to explore genes and cell types of interest. The raw RNA-Seq

data for 10 liver samples from KL mice is available in the GEO Database under the accession number GSE107470. An additional 10 samples, specifically processed for this manuscript, can be accessed under the accession number GSE286259.

Research involving human participants, their data, or biological material

Policy information about studies with human participants or human data. See also policy information about sex, gender (identity/presentation), and sexual orientation and race, ethnicity and racism.

Reporting on sex and gender	This information has not been collected.
Reporting on race, ethnicity, or other socially relevant groupings	This information has not been collected.
Population characteristics	This information has not been collected.
Recruitment	We analyzed patient records from publicly available database, including the PanCancer Atlas of The Cancer Genome Atlas (TCGA) Program and the Genotype-Tissue Expression (GTEx) Project.
Ethics oversight	*Identify the organization(s) that approved the study protocol.*

Note that full information on the approval of the study protocol must also be provided in the manuscript.

Field-specific reporting

Please select the one below that is the best fit for your research. If you are not sure, read the appropriate sections before making your selection.

☒ Life sciences ☐ Behavioural & social sciences ☐ Ecological, evolutionary & environmental sciences

For a reference copy of the document with all sections, see nature.com/documents/nr-reporting-summary-flat.pdf

Life sciences study design

All studies must disclose on these points even when the disclosure is negative.

Sample size	No statistical methods were used to pre-determine sample sizes but our sample sizes are similar to those reported in previous publications
Data exclusions	No data were excluded.
Replication	Experiments were repeated at least twice and/or reproduced using different methods (e.g. different primers, RNAi lines). Minimum three biological replicates were used in each experiment. All attempts at replication were successful.
Randomization	Samples were allocated to control and experimental groups based on their genotypes.
Blinding	Blinding is impossible because animals with different genotypes showed visible distinct phenotypes.

Reporting for specific materials, systems and methods

We require information from authors about some types of materials, experimental systems and methods used in many studies. Here, indicate whether each material, system or method listed is relevant to your study. If you are not sure if a list item applies to your research, read the appropriate section before selecting a response.

Materials & experimental systems

n/a	Involved in the study	
☐	☒	Antibodies
☐	☒	Eukaryotic cell lines
☒	☐	Palaeontology and archaeology
☐	☒	Animals and other organisms
☒	☐	Clinical data
☒	☐	Dual use research of concern
☒	☐	Plants

Methods

n/a	Involved in the study	
☒	☐	ChIP-seq
☐	☒	Flow cytometry
☒	☐	MRI-based neuroimaging

Antibodies

Antibodies used	Phospho-Stat3 (Tyr705) (D3A7) XP® Rabbit mAb Cell Signaling (#9145) (1:100), rabbit anti-pH3 from Millipore (06-570)(1:1000), Goat Anti-Rabbit IgG Antibody (H+L), Biotinylated, R.T.U. (BP-9100-50, Vector) (1:1000), donkey anti-rabbit 565 (1:2000, Molecular Probes A31572).
Validation	These antibodies were validated by the companies and has been used and referenced in many publications. Cell Signaling (#9145) is valid by the company of IHC in mouse, and cited by 4305 publications, such as PMID: 39670305.<br>Millipore (06-570) has been used and cited in many Drosophila studies, such as PMID: 29560857, and PMID: 38926577.

Eukaryotic cell lines

Policy information about cell lines and Sex and Gender in Research

Cell line source(s)	Lewis Lung Carcinoma (LLC) cell line was purchased from American Type Culture Collection (ATCC).
Authentication	LLC cells were obtained from ATCC (LL/2-LLC1; CRL-1642). As a biological resource center, ATCC performs authentication and quality-control tests on all distribution lots of cell lines. Authentication method was not specified.
Mycoplasma contamination	Tested negative for mycoplasma contamination.
Commonly misidentified lines<br>(See ICLAC register)	Based on the ICLAC Register of Misidentified Cell Lines, there are no commonly misidentified cell lines.

Animals and other research organisms

Policy information about studies involving animals; ARRIVE guidelines recommended for reporting animal research, and Sex and Gender in Research

Laboratory animals	Drosophila melanogaster, KrasG12D/+; Lkb1f/f mice (12- to 20-week-old) , C57BL/6J mice (8 weeks old).
Wild animals	This study did not involve wild animals.
Reporting on sex	Female flies are used in all experiments as they showed more significant and consistent bloating phenotype. Both male and female mice are use in the study. Sex and gender were not considered in the design of present research, and related information was not collected for the analysis of human data.
Field-collected samples	The study did not involve samples collected from the field.
Ethics oversight	The KrasG12D/+; Lkb1f/f mice experiments were approved by the Institutional Animal Care and Use Committee (IACUC) of Weill Cornell Medical College and maintained as approved by the Institutional Animal Care and Use Committee (IACUC) at Weill Cornell Medicine (NY). C57BL/6J mice experiments were approved by the Institutional Animal Care and Use Committee (IACUC) at Cold Spring Harbor Laboratory (CSHL) and were conducted in accordance with the National Institutes of Health Guide for the Care and Use of Laboratory Animals.

Note that full information on the approval of the study protocol must also be provided in the manuscript.

Flow Cytometry

Plots

Confirm that:

☒ The axis labels state the marker and fluorochrome used (e.g. CD4-FITC).

☒ The axis scales are clearly visible. Include numbers along axes only for bottom left plot of group (a 'group' is an analysis of identical markers).

☒ All plots are contour plots with outliers or pseudocolor plots.

☒ A numerical value for number of cells or percentage (with statistics) is provided.

Methodology

Sample preparation	Whole-body flies without heads were flash-frozen using liquid nitrogen and were homogenized in 1ml dounce in buffer of 250 mM sucrose, 10 mM Tris pH 8.0, 25 mM KCl, 5mM MgCl, 0.1% Triton-X, 0.5% RNasin plus (Promega, N2615), 1X protease inhibitor (Promega, G652A), 0.1 mM DTT, then filtered through 40 um cell strainer and 40 um Flowmi (BelArt, H13680-0040). Samples were centrifuged, washed, and resuspended in 1 X PBS with 0.5% BSA and 0.5% RNasin plus. The suspension was filtered again with 40 um Flowmi immediately before FACS sorting.
Instrument	Sony SH800Z Cell Sorter

Software	Sony SH800Z software
Cell population abundance	Approximately 10–20% of the retrieved nuclei were healthy. The nuclei were visually examined and confirmed under a microscope.
Gating strategy	Nuclei were stained with fluorescent DNA dye DRAQ7, multiple nuclei populations indicate polyploid cells.

☒ Tick this box to confirm that a figure exemplifying the gating strategy is provided in the Supplementary Information.
